# Telomeric autophagy in human health, aging, and diseases

**DOI:** 10.3389/fmolb.2026.1922188

**Published:** 2026-09-29

**Authors:** Xingyao Li, Xili Wu

**Affiliations:** Department of Integrated Traditional Chinese and Western Medicine, Second Affiliated Hospital of Xi’an Jiaotong University, Xi’an, Shaanxi, China

**Keywords:** aging, autophagy, diseases, telomeres, telomeric autophagy

## Abstract

Telomeres are repetitive DNA sequences that cap chromosome ends and protect genomic stability. The term “telomeric autophagy” has been applied to the relationship between telomere dysfunction and autophagy, but it has been used to describe two processes that are not equivalent, and much of the confusion in this literature follows from conflating them. The first is the induction of autophagy by dysfunctional telomeres. This is established: during replicative crisis, telomeric damage generates cytosolic chromatin fragments that activate cGAS-STING signalling and engage the autophagy machinery, an induction specific to telomeric damage and not produced by intrachromosomal breaks, and one that has been independently reproduced in cells maintaining telomeres through alternative lengthening. The second is the selective autophagic degradation of telomeric chromatin itself. For this we were unable to identify direct evidence: no receptor bridging telomeric chromatin to ATG8-family proteins has been described, telomeric cargo has not been recovered from autolysosomes, and no compound acts selectively on the process. This review therefore separates the two throughout, grades the evidence for each claim it makes, and applies the same grading to the disease literature, where cancer emerges as the only setting in which telomeric and autophagic endpoints have been measured in the same system. Its aim is to establish what is known with confidence, what rests on inference, and what would have to be shown for telomere-selective autophagy to be regarded as a pathway. Telomeric autophagy; telomere; aging; cellular senescence; autophagy; cancer; neurodegenerative diseases.

## 1 Introduction

Telomeres are repetitive DNA sequences located at the ends of chromosomes, and form complexes with associated proteins. Their primary functions are to protect chromosome ends from degradation and prevent chromosome fusion (Chakravarti et al., 2021). The length and integrity of telomeres are critical in maintaining genomic stability (Nassour et al., 2019). The gradual shortening of telomeres during cell division is a process closely associated with cellular aging. Recent studies show telomeric autophagy plays a key role in maintaining telomere integrity (Hayashi et al., 2015). This paper will systematically review the mechanisms of telomeric autophagy and its role in health, aging, and diseases.

Two lines of work converge on this topic from different directions. Telomere biology has established how chromosome ends are protected and how their deprotection is sensed; autophagy research has established how cytoplasmic material is selected and delivered to the lysosome. The point at which they meet was defined experimentally in 2019, when macroautophagy was shown to be the dominant mode of cell death during replicative crisis, and suppressing it allowed checkpoint-deficient cells to bypass crisis and accumulate genome instability (Nassour et al., 2019). What made that observation specific rather than incidental was that intrachromosomal double-strand breaks did not elicit the same response (Nassour et al., 2019). The mechanism runs through the cytosol: dysfunctional telomeres bud from the nucleus in fragile envelopes that rupture, exposing telomeric DNA to cGAS (Nassour et al., 2019). Independent work in cells using alternative lengthening of telomeres reached the same junction from the opposite direction, and found the resulting autophagy to be pro-survival rather than lethal (Barroso-González et al., 2019) — a contrast that recurs throughout this review.

Telomeric autophagy plays an important role in cellular and organismal aging. By sequestering and eliminating damaged telomeres, telomeric autophagy may delay the onset of cellular senescence. Dysfunction of telomeric autophagy has been implicated in the pathogenesis of various human diseases, including cancers, neurodegenerative diseases, cardiovascular diseases, diabetes, and liver diseases. The mechanisms underlying telomeric autophagy dysfunction vary among these conditions, involving alterations in telomere-protective proteins, mutations in autophagy-related genes, dysregulation of mTOR and p53 signaling pathways, oxidative stress, and lysosomal dysfunction.

Rather than surveying the field’s structure, it is worth stating plainly where the evidence now stands, because that is what has changed most since this topic was last reviewed. Three findings since 2019 carry the weight: the demonstration that telomere dysfunction specifically triggers autophagy through cytosolic DNA sensing (Nassour et al., 2019); the independent reproduction of that route in a different telomere-maintenance background, with the opposite functional outcome (Barroso-González et al., 2019); and the resolution of a downstream branch in which TERRA transcribed from dysfunctional telomeres binds ZBP1 and signals to mitochondria (Nassour et al., 2023). Against these, a second set of propositions has been repeated in the secondary literature without acquiring experimental support: that shelterin components interact directly with LC3 or Beclin-1, that ubiquitination of those components marks telomeres for autophagic recognition, and that agents such as rapamycin act on a telomere-directed arm of the pathway. We examined each and report what we found. Sections 2 and 3 set out the mechanism and its evidential status, Sections 4 and 5 apply the same standard to the diseases in which telomeric autophagy has been invoked, and Sections 6 and 7 treat dysfunction and intervention respectively.

### 1.1 The macroautophagy pathway: a brief overview

Because the proteins discussed throughout this review (LC3, Beclin-1, p62/SQSTM1) are components of the general autophagy machinery, we first summarise that machinery briefly. Macroautophagy (hereafter autophagy) is the principal route by which cytoplasmic material is delivered to the lysosome for degradation and recycling (Mizushima and Komatsu, 2011). It proceeds through a defined sequence: initiation, in which the ULK1 kinase complex is activated upon inhibition of mTOR or activation of AMPK; nucleation of an isolation membrane, which requires the class III phosphatidylinositol 3-kinase complex containing Beclin-1; elongation and closure of that membrane to form a double-membraned autophagosome, a step that depends on ubiquitin-like conjugation systems and culminates in the lipidation of LC3 (conversion of LC3-I to LC3-II) and its stable insertion into the autophagosomal membrane; and finally fusion of the autophagosome with the lysosome, whereupon the sequestered contents are degraded (Mizushima and Komatsu, 2011).

Autophagy may be non-selective, as during starvation-induced bulk turnover, or selective, in which a defined cargo is targeted for degradation. Selectivity is conferred by autophagy receptors such as p62/SQSTM1, which bind ubiquitinated cargo through a ubiquitin-associated domain and simultaneously bind LC3 through a short LC3-interacting region, thereby physically bridging the cargo to the nascent autophagosome. Established selective pathways, such as mitophagy, are defined by three features: a physically identified cargo, a receptor that links that cargo to ATG8-family proteins, and demonstration that clearance of the cargo depends on the core autophagy machinery. Consensus guidelines in the field recommend that any claim of a selective autophagic process be supported by genetic interference with at least two autophagy genes acting at different steps, together with assays of autophagic flux rather than steady-state marker levels alone (Klionsky et al., 2021). These criteria are referred to again in Section 2.5.

## 2 Mechanisms of telomeric autophagy

Telomeric autophagy refers to the selective degradation of telomeres through the autophagy pathway. Research indicates telomeric autophagy is essential for cellular responses to telomere damage and for regulating telomere length (Nassour et al., 2019; Hayashi et al., 2015). When telomeres are damaged or the cell is under stress, the telomere complex may be selectively degraded via autophagy, removing damaged telomeres and providing an opportunity for telomerase to extend them (Nassour et al., 2019; Jiménez-Martín et al., 2025; Zhang et al., 2021). Telomeres become substrates for this response under defined conditions rather than generally: replicative exhaustion in checkpoint-deficient cells entering crisis (Nassour et al., 2019; Hayashi et al., 2015), oxidative damage to the G-rich telomeric repeat (Opresko et al., 2025), and the replication stress that arises when the fork encounters telomeric secondary structure. The TRF2-HMGB1 effect described below was characterised under nutrient starvation and altered redox state specifically (Iachettini et al., 2023). Distinguishing these matters, because the downstream consequence is not uniform across them.

### 2.1 Role of telomere protective proteins

Telomere protective proteins, such as telomeric repeat-binding factor (TRF)1 and TRF2, play crucial roles in telomeric autophagy (Dehé et al., 2012; Markiewicz-Potoczny et al., 2021; Iachettini et al., 2023). These proteins not only regulate telomere length and protect telomeres from DNA damage responses, but may also mediate the initiation and execution of telomeric autophagy through interactions with autophagy-related proteins (Kaur et al., 2021; Feuerhahn et al., 2015).

Functions of TRF1 and TRF2: TRF1 and TRF2 bind directly to telomeric DNA, forming protective complexes that prevent telomere ends from being recognized as DNA damage (Bower and Griffith, 2014; de Lange, 2018; Walker and Zhu, 2012). A direct physical interaction between TRF1 or TRF2 and LC3 or Beclin-1 has not, to our knowledge, been demonstrated. The one molecularly defined route by which a shelterin component influences autophagy is indirect: TRF2 binds the chromatin-associated protein HMGB1 and controls its nuclear-to-cytoplasmic translocation, such that silencing TRF2 promotes HMGB1-driven autophagy, whereas overexpression of wild-type TRF2 — but not an HMGB1-binding-deficient mutant—suppresses it (Iachettini et al., 2023). This is an extratelomeric function of TRF2 and does not by itself establish autophagic degradation of telomeres. The architecture of the six-subunit shelterin complex assembled by these proteins, and the domain organisation of TRF2, are summarised in Figure 1.

**FIGURE 1:**
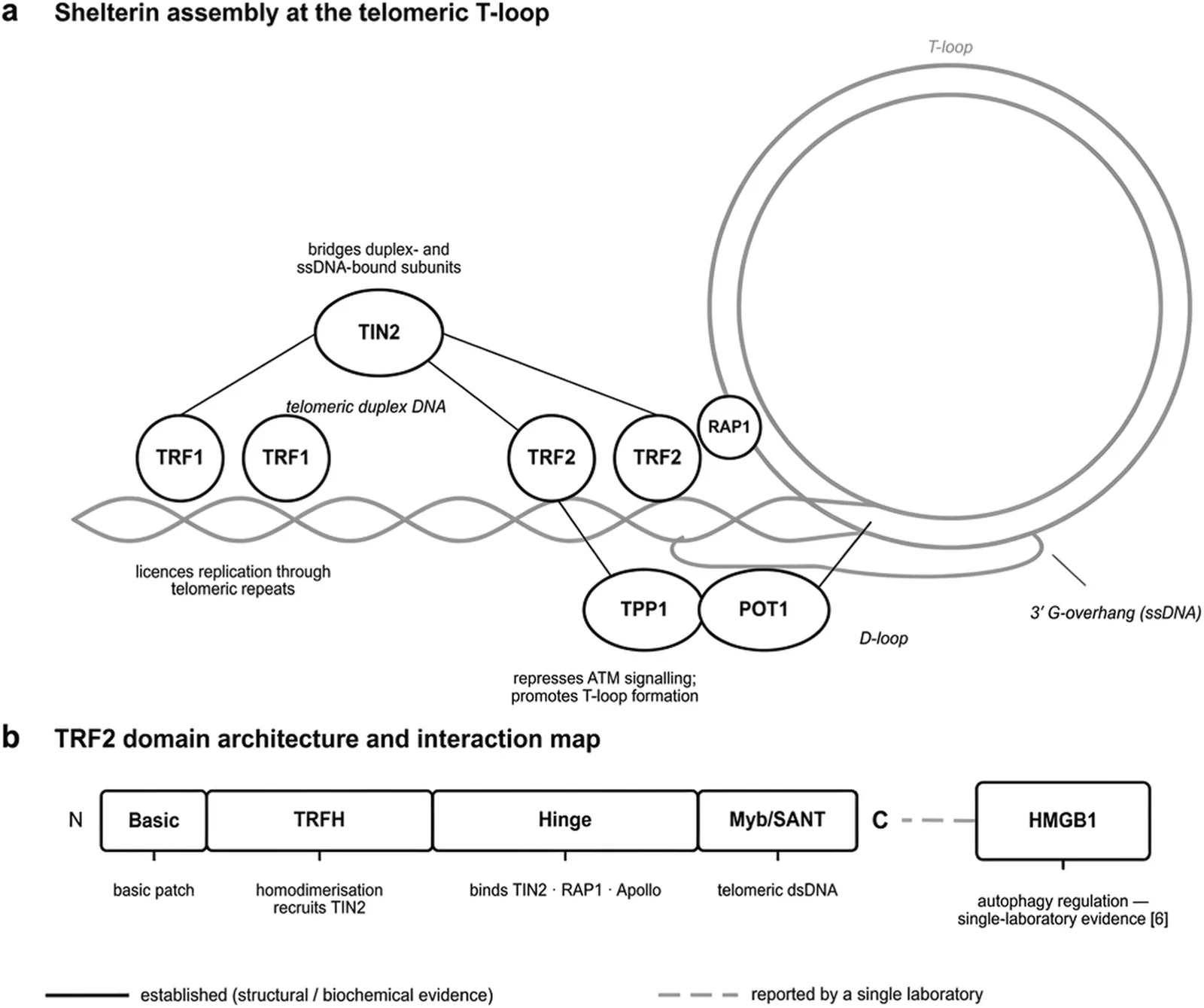
Architecture of the shelterin complex at the telomeric end (de Lange, 2018). **(a)** Assembly of the six shelterin subunits on telomeric DNA. TRF1 and TRF2 homodimers bind the double-stranded TTAGGG repeats; RAP1 associates with TRF2; TIN2 bridges the duplex-bound factors to the TPP1–POT1 heterodimer, which protects the 3′single-stranded G-overhang. TRF2 promotes invasion of the overhang into upstream duplex DNA, forming the protective T-loop, with a displacement loop (D-loop) at the invasion site. **(b)** Domain architecture of TRF2: an N-terminal basic region, the TRFH dimerisation domain that recruits TIN2, a hinge region binding TIN2, RAP1 and Apollo, and a C-terminal Myb/SANT DNA-binding domain. Solid lines denote interactions established by structural and biochemical evidence; the dash-dot line marks the TRF2–HMGB1 interaction, reported by a single laboratory to date (Iachettini et al., 2023).

### 2.2 Involvement of autophagy-related proteins

Autophagy-related proteins are also key players in telomeric autophagy. Core regulatory proteins of the autophagy process, such as LC3 (microtubule-associated protein 1A/1B-light chain 3), Beclin-1, and P62/SQSTM1, are involved in the formation, recognition, and degradation of autophagosomes (Bitto et al., 2014; Huang et al., 2023; Jeong et al., 2019).

- Role of LC3: LC3 is a hallmark protein in the autophagy process. LC3 is lipidated (converted from LC3-I to LC3-II) on the autophagosome membrane, marking the formation and maturation of autophagosomes. LC3 is required for autophagosome biogenesis generally; a telomere-specific role for LC3 has not been reported, and LC3 has not been shown to interact with telomere-binding proteins (Kondo et al., 2025).
- Role of Beclin-1: Beclin-1 is a key regulator of autophagosome formation and nucleation. Studies indicate Beclin-1 may regulate the initiation and execution of telomeric autophagy through interactions with telomere protective proteins or other autophagy-related proteins (Houtman et al., 2019; Kaur and Changotra, 2020; Li et al., 2010).
- Role of P62/SQSTM1: P62/SQSTM1 is an autophagy receptor protein that recognizes ubiquitinated proteins or organelles and transports them to autophagosomes for degradation (Clausen et al., 2010). Whether p62/SQSTM1 — or any other characterised selective autophagy receptor—recognises telomeric chromatin as cargo has not been examined directly, and we are not aware of evidence that it does.

### 2.3 Regulation of signaling pathways

Telomeric autophagy is regulated by various signaling pathways, including the mechanistic target of rapamycin (mTOR) and p53 pathways. These pathways influence the efficiency and process of telomeric autophagy by regulating critical steps in autophagy (Collins et al., 2021; Maiuri et al., 2010; Mrakovcic and Fröhlich, 2018).

- mTOR Pathway: This pathway is a key regulator of autophagy. mTOR, a serine/threonine kinase, negatively regulates autophagy by inhibiting the expressions of autophagy-related genes (ATGs) and autophagosome formation. Studies suggest inhibiting mTOR activity can activate autophagy, including telomeric autophagy. mTOR inhibitors (e.g., rapamycin) can promote telomeric autophagy, helping maintain telomere length and function (Kim and Guan, 2015; Kodali et al., 2025).
- p53 Pathway: p53 is a crucial tumor suppressor protein involved in cell cycle regulation and DNA damage response. Research shows p53 also plays a key role in telomeric autophagy. When telomeres are damaged, p53 can activate autophagy pathways, and thus promotes telomeric autophagy to maintain telomere function and cellular homeostasis (Maiuri et al., 2010; Kruse and Gu, 2009).

### 2.4 A proposed model for selective recognition: current status

The following model has been proposed in the literature but, as set out in Section 2.5, is not yet supported by direct experimental evidence; we present it here as a hypothesis to be tested rather than as an established mechanism. It has been suggested that damaged telomeric regions might first be recognized by telomere protective proteins, and then transported to autophagosomes for encapsulation and degradation through autophagy receptor proteins, such as P62/SQSTM1. Judged against the criteria by which selective autophagy pathways are normally established—a physically defined cargo, a receptor bridging it to an ATG8-family protein, and demonstrated dependence on the core machinery (Mizushima and Komatsu, 2011; Klionsky et al., 2021; Vargas et al., 2023) — this model currently satisfies none of the three.

Figure 2 summarises the elements discussed in this section and their relationship to one another. The upper panel corresponds to Section 2.1: telomeric repeat DNA at chromosome ends, bound by the shelterin components TRF1 and TRF2 together with TIN2 and POT1. The middle panel depicts telomere deprotection or damage, the trigger for the responses described in Section 2.5. The lower panels correspond to the general autophagy machinery introduced in Section 1.1, in which Beclin-1 acts at the nucleation step, LC3-II marks the elongating autophagosomal membrane, and fusion with the lysosome completes degradation. The vertical arrows between panels represent the proposed rather than the demonstrated relationship: as discussed in Section 2.5, the evidence establishes that telomere dysfunction induces autophagy, but not that telomeric chromatin is itself the autophagic cargo.

**FIGURE 2:**
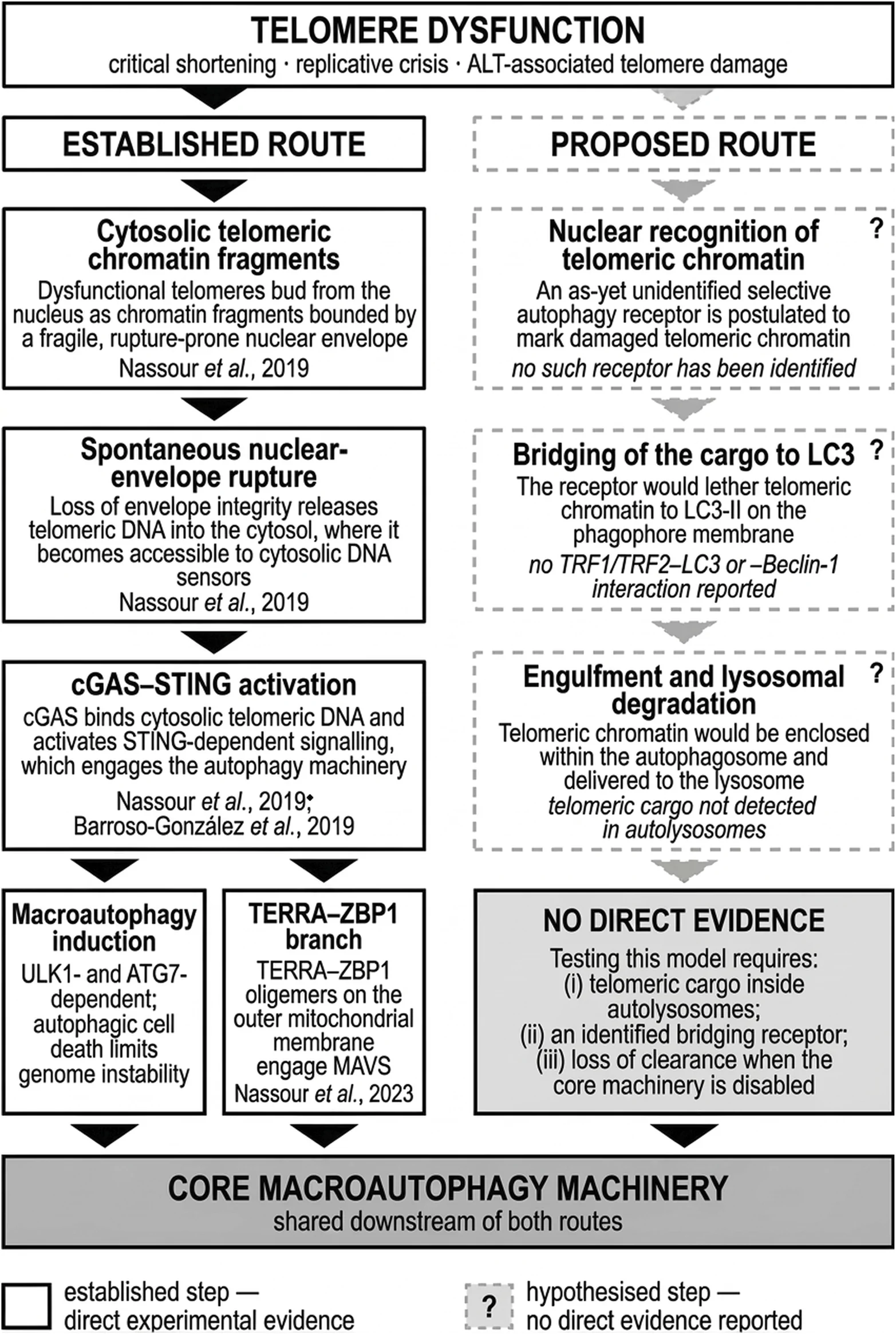
The telomere–autophagy axis, separating established steps from hypothesised ones. Left (solid outlines, solid arrows): the route for which direct experimental evidence exists. Dysfunctional telomeres bud from the nucleus as chromatin fragments enclosed by a fragile, rupture-prone nuclear envelope; envelope rupture releases telomeric DNA into the cytosol, where cGAS binds it and STING-dependent signalling engages the autophagy machinery. Two downstream branches are shown: ULK1- and ATG7-dependent macroautophagy, and the TERRA–ZBP1 branch signalling to MAVS on the outer mitochondrial membrane. Right (dashed outlines, dashed arrows, question marks): the alternative route that is frequently assumed but has not been demonstrated, in which telomeric chromatin is recognised within the nucleus by a selective autophagy receptor, bridged to LC3-II and degraded in the lysosome. Each hypothesised step is annotated with the evidence that is missing: no such receptor has been identified, no TRF1/TRF2 interaction with LC3 or Beclin-1 has been reported, and telomeric cargo has not been detected in autolysosomes. Both routes converge on the core macroautophagy machinery (bottom band), which is shared with bulk autophagy. The distinction drawn here between cytosolic telomeric DNA as a signalling trigger and nuclear telomeric chromatin as a putative cargo is developed in Sections 2.4 and 2.5.

### 2.5 Current status of the evidence and its limitations

It is useful to state plainly which aspects of the above are supported by direct evidence and which are not. Direct evidence exists that telomere dysfunction induces autophagy. During replicative crisis, macroautophagy is the dominant mode of cell death in fibroblasts and epithelial cells, and suppressing autophagy allows cells to bypass crisis and accumulate genome instability; notably, this induction is specific to telomeric damage, as intrachromosomal double-strand breaks did not elicit the same response (Nassour et al., 2019). Mechanistically, telomeric DNA damage generates cytosolic chromatin fragments that activate the cGAS-STING pathway, which then engages the autophagy machinery (Nassour et al., 2019). This route has been independently corroborated in cells using alternative lengthening of telomeres, where cytosolic telomeric DNA was sensed by cGAS and activated ULK1- and ATG7-dependent autophagy (Barroso-González et al., 2019), and a downstream branch involving TERRA-bound ZBP1 signalling to mitochondria has since been described (Nassour et al., 2023).

By contrast, we did not identify direct evidence for the selective autophagic degradation of telomeric chromatin itself. Specifically, no physical interaction between TRF1 or TRF2 and LC3 or Beclin-1 has been reported; none of the characterised selective autophagy receptors has been shown to recognise telomeric cargo. This includes p62/SQSTM1, NBR1 and optineurin (OPTN), the receptors through which ubiquitinated cargo is bridged to ATG8-family proteins in established selective pathways (Vargas et al., 2023; Li W. et al., 2021; Liu et al., 2024); we found no study that tested any of them against telomeric chromatin; and no study has demonstrated that clearance of telomeric material depends on the core autophagy machinery using the genetic criteria recommended for such claims (Klionsky et al., 2021). Much of the existing literature also relies on steady-state markers such as LC3-II levels, which can increase either because autophagy is induced or because autophagosome-lysosome fusion is blocked, and therefore cannot on their own establish increased telomere-directed autophagic flux (Klionsky et al., 2021). A further limitation is that the human data linking telomeres to age-related disease derive almost entirely from leukocyte telomere length, which is an imperfect proxy for telomere status in the affected tissue. We therefore treat telomere-selective autophagy as an emerging hypothesis rather than an established pathway, and note that testing it will require demonstrating telomeric cargo within autolysosomes, identifying a bridging receptor, and showing that clearance is lost upon interference with the core machinery.

It is also worth being explicit about what the principal experimental approaches in this area can and cannot establish. Co-localisation imaging, which places telomeric signal in proximity to autophagosomal or lysosomal markers, is necessarily static: proximity at a fixed time point does not distinguish selective capture of telomeric chromatin from the incidental presence of telomere-derived fragments among bulk cytoplasmic cargo, and it cannot establish the direction or the kinetics of transfer. Genetic interference with autophagy genes, followed by rescue, is the approach that can establish dependence, and consensus guidelines recommend targeting at least two genes acting at different steps of the pathway (Klionsky et al., 2021); to our knowledge this design has not been applied with telomeric endpoints. The cGAS-STING models used in the crisis studies (Nassour et al., 2019; Barroso-González et al., 2019) are informative precisely because they combine genetic perturbation with defined telomeric triggers, but their readout is the induction of autophagy rather than the fate of telomeric DNA. Distinguishing these possibilities will require following telomeric sequence into the autolysosome directly.

Coupling between the telomeric DNA damage response and the autophagy machinery. The DNA damage response (DDR) arm of this relationship is well characterised; its connection to autophagy is not, and the two should not be stated with equal confidence. Deprotected telomeres recruit γH2AX and 53BP1 into telomere dysfunction-induced foci (TIF), first quantified in human chronic lymphocytic leukaemia cells with resected telomeric 3′overhangs (Brugat et al., 2010) and now scored routinely by proximity ligation in human and mouse cells (Wang et al., 2022). TIF burden rises whenever shelterin is depleted—after miR-23a-mediated loss of TRF2 (Luo et al., 2015) or miR-185-mediated loss of POT1 (Li et al., 2020)—and in both cases accelerates replicative senescence in primary human fibroblasts. Evidence that this telomeric DDR then engages autophagy is far thinner and rests largely on G-quadruplex ligands. SYUIQ-5 delocalised TRF2 from telomeres, generated telomeric γH2AX foci, activated ATM and induced LC3-II; the ATM inhibitor KU-55933 and ATM siRNA each reduced γH2AX and LC3-II together, and ATG5 knockdown blunted the resulting cytotoxicity (Zhou et al., 2009). To our knowledge this is the only report placing ATM upstream of autophagy in an explicitly telomeric damage context, and it comes from a single laboratory using cancer cell lines. Extrapolation beyond it is unreliable in both directions: a second G-quadruplex ligand activated the same ATM–autophagy axis while producing global but expressly no telomeric damage (Beauvarlet et al., 2019); telomere-homologous T-oligo induced autophagy with ATM/ATR signalling dispensable (Rankin et al., 2012); and dihydroartemisinin elicited p-ATM, p-ATR and γH2AX alongside autophagy that was explicitly uncoupled from senescence and cell death (Zou et al., 2019). General DDR-to-autophagy mediators such as DRAM1 (Yang J. et al., 2025), and the wider crosstalk between repair and autophagic pathways (Demirbağ-Sarikaya et al., 2021), have not been interrogated at telomeres. We therefore treat ATM as a plausible but singly-sourced coupling node, and the remainder of the DDR–autophagy interface as inference from non-telomeric systems.

### 2.6 Comparison with established selective autophagy pathways

The distinction drawn in Section 2.5 becomes clearer when the telomere case is set alongside the selective autophagy pathways that are regarded as established (Table 1). For mitophagy and ER-phagy, all three of the criteria introduced in Section 1.1 are satisfied: the cargo is physically defined, a receptor bridging that cargo to ATG8-family proteins has been identified, and clearance has been shown to depend on the core machinery (Chino et al., 2019; Vargas et al., 2023). Ribophagy, nucleophagy and aggrephagy are recognised as selective processes and are catalogued as such in current reviews (Vargas et al., 2023; Li W. et al., 2021), although the receptors and mechanisms are less completely resolved in mammalian systems than for mitophagy. Selective autophagy of several of these cargoes has been implicated in cancer (Liu et al., 2024), and chaperone-mediated autophagy provides a further example of selective lysosomal targeting operating through an entirely different route (Kaushik and Cuervo, 2018).

**TABLE 1:** Comparison of selective autophagy pathways. Rows are ordered by completeness of the supporting evidence. The final row summarises the status of telomere-selective autophagy: every cell reads “no direct evidence identified”, which is the principal observation of this review rather than an omission. Criteria for regarding a selective pathway as established are given in Section 1.1 and are applied to the telomere case in Section 2.5.

Pathway	Cargo	Receptor	Core mechanism	Disease involvement	Evidence status
Mitophagy	Damaged mitochondria	PINK1–Parkin axis; BNIP3/NIX (Vargas et al., 2023; Li D. et al., 2021)	Ubiquitin- and receptor-mediated capture via LIR–ATG8 binding (Vargas et al., 2023)	Neurodegeneration, cancer, cardiometabolic disease (Li D. et al., 2021; Liu et al., 2024)	Established
ER-phagy	Endoplasmic reticulum fragments	TEX264, identified by LIR-dependent screen (Chino et al., 2019)	Receptor LIR binds LC3/GABARAP; intrinsically disordered region bridges membranes (Chino et al., 2019)	Catalogued among selective pathways in cancer (Liu et al., 2024)	Established
Ribophagy	Ribosomes	Reported; less fully resolved in mammals (Vargas et al., 2023)	Selective capture of ribosomal cargo (Vargas et al., 2023)	Listed among organelle-selective routes (Li D. et al., 2021; Liu et al., 2024)	Recognised; mechanism incomplete
Nucleophagy	Nuclear components	Reported; less fully resolved in mammals (Li D. et al., 2021)	Selective degradation of nuclear material via the lysosomal system (Li D. et al., 2021)	Listed among organelle-selective routes (Liu et al., 2024)	Recognised; mechanism incomplete
Aggrephagy	Large protein aggregates	p62/SQSTM1 and related ubiquitin-binding receptors (Vargas et al., 2023; Li D. et al., 2021)	Ubiquitin-tagged aggregates bridged to ATG8 proteins (Vargas et al., 2023)	Protein-aggregation disorders (Li D. et al., 2021)	Established
Telomere-selective autophagy (hypothetical)	No direct evidence identified	No direct evidence identified	No direct evidence identified	No direct evidence identified	Hypothesis; not tested against the criteria applied to the rows above

The telomere row of Table 1 is empty in every column. This is the substantive point of the comparison, and we would rather state it plainly than present it as a gap awaiting routine filling. No telomeric cargo has been recovered from autolysosomes; no receptor bridging telomeric chromatin to ATG8-family proteins has been identified, and the shelterin components most often proposed for that role have not been shown to bind LC3 or Beclin-1; no dependence of telomeric clearance on the core machinery has been demonstrated; and no modulator acting selectively on this process exists. Telomere-selective autophagy is therefore best described not as an under-studied pathway but as a hypothesis that has not yet been tested against the standards routinely applied to the other rows of this table. Establishing it would require, at minimum, direct demonstration of telomeric sequence within autolysosomes, identification of a protein carrying a functional LC3-interacting region that associates with telomeres, and loss of telomeric clearance upon interference with at least two core autophagy genes acting at different steps (Klionsky et al., 2021).

The reviewer asks how different forms of selective autophagy operate within a single disease. Cancer is the setting in which this can be answered most fully: mitophagy, ER-phagy, ribophagy and nucleophagy have all been implicated in tumour biology, and current reviews treat them as parallel quality-control routes whose relative contributions differ by tumour type and metabolic context (Liu et al., 2024). Neurodegeneration is a second such setting, where mitochondrial and aggregate clearance are both compromised (Vargas et al., 2023; Li W. et al., 2021). What these examples share is that each pathway is defined by its own cargo and receptor, and that their disease contributions are established separately rather than inferred from one another. That is precisely the standard telomere-selective autophagy has not yet met, and it is why we do not assign it a role alongside the others in any disease discussed in this review.

- Autophagosome Encapsulation: Once the damaged telomeric regions are recognized, they are encapsulated into autophagosomes via autophagy receptor proteins, such as P62/SQSTM1. The LC3 protein plays a key role in this process by facilitating the formation and maturation of the autophagosome membrane through its lipidation.

## 3 Telomeric autophagy and aging

Aging is a series of degenerative changes in biological function that occur over time. Telomere shortening is recognized as one key marker of cellular aging. Telomeric Autophagy, which regulates telomere length and maintains telomere function, plays a potential role in delaying cellular aging (Li Y. et al., 2024).

### 3.1 Telomere length and cellular aging

Telomeres are repetitive DNA sequences at the ends of chromosomes, and are associated with protective proteins. These structures form a telomere-protective complex that prevents chromosome ends from being mistaken as damaged DNA and being repaired. In each cell division, telomeres gradually shorten due to limitations in the DNA replication mechanism. Once they reach a critical length, cells enter a state of senescence or undergo apoptosis (Drury et al., 2012; Willeit et al., 2010).

Telomere shortening is closely linked to the decline in the replication capacity of a cell. Shortened telomeres activate the DNA damage response, causing cell cycle arrest and ultimately leading to senescence or apoptosis. Cellular senescence is a permanent state of cell cycle arrest, and is characterized by weakened response to growth factors, altered gene expression, and diminished metabolic activity (Chakravarti et al., 2021; Li Y. et al., 2024).

Telomeres shorten at every round of replication, and once a telomere falls below a critical length its protective conformation is lost and the chromosome end is registered as damaged DNA; the ensuing damage response enforces the permanent cell-cycle arrest of replicative senescence, or apoptosis (Savage, 2024), (Coulter et al., 2024). Work since 2023 has made this account more specific in two respects. Telomere length is not a single quantity per cell: nanopore profiling of 147 individuals resolved length at near single-nucleotide resolution and showed that individual chromosome ends carry consistently different lengths, differing by more than six kilobases, with the same rank order already present in newborn cord blood and preserved as telomeres shorten with age (Karimian et al., 2024). Sequencing at up to 30-bp resolution further showed that human ageing is accompanied by a progressive loss of long telomeres and an accumulation of short ones, and that in patients with genetic defects in telomere maintenance this accumulation is more pronounced and correlates with phenotypic severity (Sanchez et al., 2024). Because arrest is triggered once a telomere becomes critically short rather than by the average across chromosome ends (Savage, 2024), these distributions are more informative than the mean values that clinical studies generally report. The limits of the model deserve equal precision. Among 555 community-dwelling Chinese adults aged 75 years and over, telomere length was not associated with multimorbidity, diabetes, stroke, cardiovascular disease, cognitive impairment, frailty or sarcopenia (Yeung et al., 2023), and the proposal that telomere attrition is the one causal hallmark from which the others follow (Huang and Hu, 2025) remains a hypothesis rather than a demonstrated ordering.

### 3.2 Autophagy and cellular aging

Autophagy is a process that maintains cellular homeostasis by degrading and recycling damaged or excessive cellular components under stress conditions. Its role in cellular aging has been widely studied (Zhang, 2013). A decline in autophagic function is strongly associated with aging and age-related diseases. Through autophagy, cells can remove damaged organelles and proteins, and can be further prevented from decline in cellular function (Guo J. et al., 2022; Zhang and Cuervo, 2008).

As organisms age, autophagic activity is weakened, leading to the accumulation of damaged components in cells and impairing cell function. This decline in autophagic function is considered a key mechanism of aging. Thus, enhancing autophagy can delay cellular aging and improve cellular function (Revuelta and Matheu, 2017).

Autophagic capacity falls with age, and the decline is best characterised for chaperone-mediated autophagy (CMA), which degrades individually selected cytosolic proteins after translocating them across the lysosomal membrane. CMA activity decreases with age in multiple rodent tissues and in human cell types, and the principal cause is loss of the CMA receptor LAMP2A from the lysosomal membrane (Jafari et al., 2024). Systematic profiling in CMA reporter mice, combined with single-cell transcriptomic data, showed that most organs and cell types lose CMA activity with age, that the decline is greater in males, and that it is accompanied by fewer CMA-competent lysosomes and by transcriptional downregulation of CMA genes (Khawaja et al., 2025a). The loss is not merely correlative: blocking CMA induces cellular senescence through the calcium dysregulation caused by accumulation of its substrate PLCG1, in human intervertebral disc specimens reduced LAMP2A and raised PLCG1 accompany disc senescence, and overexpression of Lamp2a suppresses disc degeneration in rats (Cheng et al., 2025). Macroautophagy behaves comparably in the nervous system, where inhibition induces neuronal and glial senescence and where disease-associated proteins in turn impair the flux that would otherwise clear them (Palmer et al., 2025). Intervention studies converge on the same node, though almost entirely in animals: caloric restriction and calorie-restriction mimetics constitutively activate CMA in old rodent liver by stabilising LAMP2A at the lysosomal membrane (Jafari et al., 2024), and genetic or pharmacological CMA activation in old mice and in a mouse model of Alzheimer’s disease restores synaptic protein levels and normalises neurotransmission (Khawaja et al., 2025b). Human intervention data are far thinner; among agents tested clinically, the rapamycin analogue everolimus improved influenza-vaccine antibody titres in older adults (Kritchevsky and Cummings, 2025).

### 3.3 Relationship between telomeric autophagy and cellular aging

Telomeric Autophagy selectively degrades damaged telomeres, and thus are potentially pivotal in delaying cellular aging. The three key points of the relationship between telomeric autophagy and aging are shown below.

Telomere Protection and Aging Delay: Telomere length is influenced by diet and metabolic state (De la Fuente et al., 2025), and autophagic capacity declines with age (Revuelta and Matheu, 2017; Kaushik et al., 2021). One study in men with obesity reported both shorter sperm telomeres and altered expression of autophagy-related genes in the same subjects (Raee et al., 2023). Co-measurement of this kind establishes association but not dependence; whether reduced autophagy accelerates telomere attrition remains untested, as no study we identified interfered with the autophagy machinery and asked whether the telomere phenotype was lost.

Population data linking telomere length to clinical outcomes are extensive, but three features must be stated before summarising them: the measurements are almost always of leukocyte telomere length (LTL) in peripheral blood rather than of telomeres in the affected tissue, the designs are observational, and the estimates are therefore associations rather than causal effects. In the largest cohort, 472,432 UK Biobank participants followed for a mean of 12 years, a one-standard-deviation reduction in LTL was associated with all-cause mortality (hazard ratio [HR] 1.08, 95% CI 1.07–1.09) and more strongly with respiratory (1.40, 1.34–1.45), musculoskeletal (1.51, 1.35–1.92) and cardiovascular (1.09, 1.06–1.12) mortality, but not with cancer mortality (Schneider et al., 2022). A meta-analysis of 25 studies (121,749 individuals, 21,763 deaths) gave a per-standard-deviation HR of 1.09 (1.06–1.13), with a 26% higher hazard (15%–38%) in the shortest versus the longest quartile and attenuation above age 80 (Wang et al., 2018). For cardiometabolic endpoints, a 1-SD decrease was associated with stroke (odds ratio 1.21, 1.06–1.37), myocardial infarction (1.24, 1.04–1.47) and type 2 diabetes (1.37, 1.10–1.72), with substantial heterogeneity (I^2^ 61%–91%) (D'Mello et al., 2015). An umbrella review of 21 meta-analyses covering 50 outcomes graded the evidence high for only one association, short telomeres and gastric cancer (relative risk 1.95, 1.68–2.26), moderate for diabetes and Alzheimer’s disease, weak for 20 outcomes and non-significant for 27 (Smith et al., 2019). The direction is not uniform: among 26,540 Singapore Chinese adults, the longest LTL decile carried a 26% higher total cancer incidence (11%–44%) than the shortest (Samavat et al., 2021). That blood is an imperfect proxy for the diseased organ is directly demonstrable: across six studies of idiopathic pulmonary fibrosis (622 patients, 544 controls) telomeres were shorter in patients (standardised mean difference −0.84, −1.21 to −0.48), with steeper shortening in lung tissue than in peripheral blood (Fachrucha et al., 2026). Three features of the disease-specific cohorts qualify how far this biomarker can be pushed. The underlying age-telomere relationship is detectable across racially diverse pulmonary fibrosis cohorts and their controls (Adegunsoye et al., 2023); in chronic kidney disease, leukocyte telomere length has been assessed alongside composite biological-age algorithms in 23,435 UK Biobank participants followed for a median of 12 years (Xiang et al., 2024); and in type 2 diabetes, both baseline and 4-year change in relative telomere length were measured in 819 participants of a community-based cohort (Yang C. et al., 2025). Designs of the latter kind are necessary because a single measurement captures neither trajectory nor tissue of origin. Whether the association is causal has been addressed directly using Mendelian randomisation together with polygenic risk scores (Cao et al., 2020), and the genetic evidence does not support the causal reading that the observational literature invites.

*In vivo* evidence in aging: three questions that cannot yet be answered. Three specific questions arise here, and each is answered in the negative. (i) Does tissue-specific enhancement of telomere-directed autophagy delay aging *in vivo*? No such experiment has been reported. Every tissue-specific autophagy manipulation performed in aged animals targets general autophagy: cardiac-specific Atg7 deletion causes senescent cardiomyocytes to accumulate and impairs cardiac function in mice, which the senolytic ABT-263 reverses (Zhai et al., 2024); muscle-specific overexpression of TRP53INP2 prevents sarcopenia in aged mice (Sebastián et al., 2024); and systemic autophagy inhibition followed by restoration gives a segmental reversal of murine aging at the cost of increased spontaneous tumours (Cassidy et al., 2020). The one tissue-specific intervention performed in a telomere-driven model—cardiac PGC1α knock-in in third-generation Terc^−/−^ mice, which restored mitochondrial homeostasis, attenuated the SASP and extended health span, yet accelerated cardiac aging in wild-type mice (Zhu et al., 2019)—targets mitochondrial biogenesis, not autophagy at telomeres. (ii) Which tissues show the steepest decline of telomeric autophagy during physiological aging? Unknown, because telomere-directed autophagy has never been measured in any tissue. What has been mapped is either general autophagy—longitudinal *in vivo* profiling of the aging mouse brain argues against a widespread decline in mitophagy and shows region- and cell-type-specific fluctuation instead (Rappe et al., 2024), TP53INP2 falls in aged mouse and human skeletal muscle (Sebastián et al., 2024), and autophagy is retained in aged rat triceps while mitophagy declines in gastrocnemius (Capitanio et al., 2016)—or telomere dysfunction alone, quantified most clearly in bone, where osteocytes from 24-month-old mice carry sixfold more TIF than those from 6-month-old mice (Farr et al., 2016). The two have never been measured in the same tissue. (iii) What is the quantitative relationship between telomeric autophagy activity and senescence markers? None has been reported. The closest direct test is *in vitro* and negative: acute telomere dysfunction rapidly induced autophagic flux in human fibroblasts, yet inhibiting autophagy changed neither the transition to senescence nor the frequency of chromosome fusions (Mar et al., 2015). Mouse studies reporting telomere length and autophagy markers together—acacetin in D-galactose-treated hearts (Hong et al., 2021), Ganoderma triterpenoids in brain (Zeng et al., 2021)—co-measure without establishing dependence, and in Terc^−/−^; 5xFAD mice the demonstrated direction is the reverse, senescence impairing autophagic flux while telomere attrition tracks neuronal loss (Suelves et al., 2023). All three questions are therefore unanswerable at present, and saying so is the most defensible conclusion the evidence supports.

Autophagy and Telomere Repair: Telomeric Autophagy not only clears damaged telomeres, but also may provide opportunities for telomerase to extend telomeres. Telomerase is capable of lengthening telomeres, though its activity is tightly regulated in somatic cells. By removing damaged telomeres, telomeric autophagy creates a better environment for telomerase to function, and thus aids in telomere repair and extension.

Regulation of Aging-Related Pathways: The mTOR and p53 pathways link telomere status to autophagy and are described in Section 2.3; the shelterin components involved are described in Section 2.1 and are not repeated here (Barja, 2019; Harris and Cheng, 2016).

### 3.4 Role of telomeric autophagy in tissue and organ aging

Telomeric Autophagy impacts aging not only at the cellular level, but may also at the tissue and organ levels (Kaushik et al., 2021; Rastaldo et al., 2020). Its three potential roles in key tissues and organs are shown below.

Skin: Skin is a critical barrier between the external environment and internal organs. Skin ageing involves multiple processes including pigmentary change (Lee, 2021), and telomere shortening has been reported in skin cells. We are not aware, however, of evidence that autophagy of telomeres occurs in skin or that enhancing it slows skin ageing, and the earlier statement to that effect has been removed.

Cardiovascular System: Telomere shortening is considered a significant risk factor for cardiovascular diseases. Effective telomeric autophagy in cardiovascular cells can slow telomere shortening and improve cellular function, helping prevent the development of cardiovascular diseases.

Nervous System: The telomere length and function of neural cells are vital for the health of the nervous system. Studies demonstrate that a decline in autophagic function is closely related to the development of neurodegenerative diseases. Telomere-associated autophagy can be regulated to clear damaged telomeres, reducing neural cell apoptosis and maintaining nervous system function (Cheon et al., 2019).

## 4 Telomeric autophagy and diseases

Telomeric Autophagy plays an essential role in maintaining telomere length and function, and its dysfunction may contribute to the onset and progression of various diseases. This section will explore the roles of telomeric autophagy in cancers, neurodegenerative diseases, cardiovascular diseases, and other related conditions.

A Unifying Framework for Understanding Disease Phenotypes. It is useful to classify diseases associated with telomeric autophagy based on their modes of autophagy dysfunction. The diseases discussed in this review can be divided into two categories: (1) Diseases with reduced autophagic activity, including neurodegenerative diseases, cardiovascular diseases, diabetes, and liver diseases, in which impaired telomeric autophagy results in the accumulation of damaged telomeres and accelerated cellular senescence (Guo J. et al., 2022; Kaushik et al., 2021); and (2) Diseases with dysregulated autophagic activity, primarily cancers, in which telomeric autophagy is either suppressed or co-opted by tumor cells depending on the stage of cancer progression (Nassour et al., 2019).

The following sections examine each disease category in detail, highlighting both the shared mechanisms and disease-specific features of telomeric autophagy dysfunction.

### 4.1 Telomeric autophagy and cancers

Cancer cells often maintain telomere length through multiple mechanisms, and thereby can evade cellular senescence and apoptosis (Chakravarti et al., 2021). Telomeric Autophagy in cancers primarily affects telomere length and function regulation.

The Dual Role of Telomeric Autophagy in Cancer. Telomeric autophagy plays a context-dependent dual role in cancer, functioning as either a tumor suppressor or a tumor promoter. During the early stages of tumorigenesis, telomeric autophagy serves as an essential tumor-suppressive barrier. When cells undergo telomere crisis induced by critically shortened telomeres, autophagy induced by the cGAS-STING innate immune pathway mediates the elimination of genomically unstable cells, preventing malignant transformation (Nassour et al., 2019). Studies have shown that autophagy-deficient cells can bypass telomere crisis with a comprehensive profile of chromosomal abnormalities, including nonreciprocal translocations and deletions, which are characteristic of epithelial cancers (Nassour et al., 2019). In established tumors, however, telomeric autophagy may switch to promote cancer progression. Cancer cells can utilize autophagy to survive in the nutrient-deprived tumor microenvironment, thereby conferring therapeutic resistance and facilitating metastasis (Barroso-González et al., 2019).

The Critical Role of p53. The outcome of telomeric autophagy in cancer is largely dependent on the status of the p53 tumor suppressor pathway. In cells with functional p53, dysfunctional telomeres induce p53-mediated cellular senescence or apoptosis, which provides a strong tumor-suppressive function (Hayashi et al., 2015; Maiuri et al., 2010; Mrakovcic and Fröhlich, 2018). In contrast, when p53 is inactivated through genetic or epigenetic alterations, dysfunctional telomeres lead to different outcomes. In p53-deficient cells, dysfunctional telomeres induce genomic instability rather than cell death. This may explain why p53 mutations are commonly found in human cancers and why loss of p53 creates a permissive environment for the survival and proliferation of genomically unstable cells (Kruse and Gu, 2009).

Reconciling the Paradox: The apparent contradiction between tumor-suppressive and tumor-promoting functions of telomeric autophagy can be reconciled by considering the temporal and contextual factors involved. During early cancer development, telomeric autophagy functions primarily as a tumor suppressor by eliminating pre-cancerous cells with critically short telomeres and genomic instability (Nassour et al., 2019). However, once cancer cells have acquired sufficient genetic alterations to bypass cell death mechanisms, telomeric autophagy may be co-opted to support tumor survival. Additionally, the specific tumor type, telomerase activity status, and the presence of alternative lengthening of telomeres (ALT) mechanisms all influence how telomeric autophagy affects cancer progression (Barroso-González et al., 2019). Understanding this dual nature is essential for developing effective therapeutic strategies that target telomeric autophagy in cancer treatment.

- Telomerase Activation: Cancer cells activate telomerase to extend telomeres, and thereby can achieve unlimited proliferation (Chakravarti et al., 2021). Whether autophagy of damaged telomeres creates a more permissive environment for telomerase has been suggested but, to our knowledge, has not been tested experimentally.
- Autophagy Inhibition and Cancer Progression: TRF2 has been shown to inhibit autophagy: it restrains HMGB1-driven autophagic activity (Iachettini et al., 2023), and its overexpression in triple-negative breast cancer increased sensitivity to taxane-based therapy through autophagy inhibition, in cell, mouse and patient-cohort analyses (Iachettini et al., 2024). We note that these studies concern autophagy in general rather than a telomere-directed process.

#### 4.1.1 New strategies for cancer treatment

Targeting telomeric autophagy can offer new strategies for cancer therapy.

- Modulating Autophagy as a Therapeutic Approach: Restoring or enhancing autophagy may induce telomere shortening and apoptosis in cancer cells; this remains a proposal rather than a demonstrated strategy, as no compound acting selectively on telomere-associated autophagy has been described.
- Combination Therapy: Combining telomeric autophagy regulators with other anticancer treatments may improve therapeutic efficacy. For instance, telomeric autophagy activators combined with chemotherapy or radiotherapy can enhance cancer cell sensitivity to treatment. (Iachettini et al., 2024)

### 4.2 Telomeric autophagy and neurodegenerative diseases

Neurodegenerative diseases, such as Alzheimer’s and Parkinson’s diseases, are closely related to cellular aging and telomere shortening (Cheon et al., 2019). A decline in autophagic function is well documented in these disorders (Kodali et al., 2025; Cheon et al., 2019). It should be noted, however, that the human data linking telomeres to neurodegeneration derive from leukocyte telomere length rather than from neural tissue, and that neurons are post-mitotic and therefore do not undergo replication-dependent telomere shortening. We are not aware of studies demonstrating that autophagy of telomeres occurs in neurons or that enhancing it reduces neuronal loss; the two literature have not been experimentally connected.

Two features of the human evidence deserve emphasis. First, it is inconsistent in direction. In the Rotterdam Study, the relationship between leukocyte telomere length and incident Alzheimer’s disease was U-shaped, with both short and long telomeres associated with increased risk (Fani et al., 2020); and in the Alzheimer’s Disease Neuroimaging Initiative cohort, longitudinal change in telomere length was not associated with diagnosis or progression (Nudelman et al., 2019). A body of evidence of this shape is difficult to reconcile with a simple model in which telomere attrition drives neurodegeneration. Second, essentially all of these measurements are made in blood. Because neurons are post-mitotic and do not undergo replication-dependent telomere shortening, leukocyte telomere length is better understood as an index of haematopoietic and immune ageing than as a proxy for the telomere state of neurons (Konopka, 2025).

Disease specificity can be supplied at the level of the autophagic machinery itself. Aβ and tau are both handled by the autophagy–lysosome pathway: tau is cleared by the proteasome and by macroautophagic and endolysosomal routes, and its post-translational modifications, aggregation state and mutations determine which route operates, generating a self-reinforcing cycle in which impaired degradation and tau pathology aggravate one another (Tang et al., 2019), (Wang L. et al., 2025). Aβ and phosphorylated tau in turn lower the levels of several autophagy and mitophagy proteins and engage in abnormal interactions with DRP1 and with PINK1–Parkin, compromising removal of damaged mitochondria (Reddy and Oliver, 2019). The lesion in AD neurons has been described as an imbalance rather than a simple deficit: autophagy induction is heightened while lysosomal degradative capacity falls, so undegraded substrates—including APP-βCTF, an inhibitor of lysosomal acidification, and membrane-damaging Aβ—build up inside lysosomes, culminating in lysosomal membrane permeabilization and cathepsin leakage (Nixon, 2024). The response is also cell-type specific. In mice, Aβ transiently induces LC3B and sustains SQSTM1 transcription in astrocytes, and astrocyte-specific knockdown of either gene increases plaque burden in APP/PS1 animals (Kim et al., 2024), whereas MTORC1 inhibition, but not AMPK activation, lowers Aβ secretion from APP/PSEN1 astrocytes (García-Juan et al., 2024); microglial autophagy is required for engagement with plaques, and its loss both aggravates neuropathology and drives microglia into a p21-positive senescent state (Choi et al., 2023). Restoring lysosomal cathepsin activity and mitophagy improves cognition in AD mice (Hou et al., 2024). Every readout in this literature, however, is Aβ or tau burden, LC3-II, SQSTM1, lysosomal pH or mitophagic flux; none measures telomere length, telomere dysfunction-induced foci or cytosolic telomeric DNA. Whether Aβ or tau modifies telomere dysfunction-induced autophagy is therefore untested, and answering it would require quantifying telomeric DNA damage and cytoplasmic telomeric fragments in Aβ- and tau-burdened neurons and glia.

Where telomere biology and autophagy do plausibly meet in the nervous system, the connection runs through the non-canonical activities of telomerase rather than through telomere length. TERT has functions outside telomere maintenance, including within mitochondria, and these have been proposed to contribute to neuronal protection (Saretzki, 2022). Similarly, SIRT6 has been described as regulating telomere homeostasis and autophagy in parallel, which places the two processes downstream of a shared regulator rather than in a linear relationship (Lu et al., 2025). Neither observation demonstrates autophagic turnover of telomeres. We are not aware of any study that has measured telomeric and autophagic endpoints in the same neural system, and the assertion that enhancing telomere-directed autophagy protects neurons is, at present, without experimental support.

#### 4.2.1 Potential therapeutic strategies for neurodegenerative diseases

Improving telomeric autophagy efficiency may be a new approach for treating neurodegenerative diseases.

- Autophagy Activators: Certain autophagy regulators, such as rapamycin and metformin, can potentially slow the progression of neurodegenerative diseases by activating autophagy pathways (Kim and Guan, 2015; Kulkarni et al., 2020). Whether these agents act on telomeres through autophagy has not been established.
- Gene Therapy: Modulating autophagy-related genes through gene therapy has been proposed as an approach in neurodegenerative disease. We note that the earlier phrase “telomeric ATGs” is not an established category; no autophagy-related gene has been shown to act specifically at telomeres.

### 4.3 Telomeric autophagy and cardiovascular diseases

Cardiovascular diseases are one of the leading causes of death worldwide. Telomere shortening is considered a major risk factor for cardiovascular diseases (Willeit et al., 2010), although this association derives from observational cohorts measuring leukocyte telomere length and does not establish causality. Autophagy, and selective autophagy in particular, is important for cardiometabolic homeostasis (Jeong et al., 2019). As in neurodegeneration, we are not aware of studies measuring telomeric and autophagic endpoints in the same cardiovascular system, and a telomere-directed autophagic process in cardiovascular cells has not been demonstrated.

The epidemiological picture is more heterogeneous than is often acknowledged. Leukocyte telomere length was not associated with coronary artery calcium in Black participants, in contrast to the association reported in other groups (Hunt et al., 2015); in the PESA cohort, associations with subclinical atherosclerosis did not persist after adjustment (Fernández-Alvira et al., 2016); and a UK Biobank analysis found that modifiable traits and health behaviours together explained less than 0.2% of the variance in leukocyte telomere length and did not alter its relationship with disease (Bountziouka et al., 2022). These findings temper the interpretation of telomere length as a modifiable cardiovascular risk factor.

Experimental work in the heart has, on several occasions, observed telomere and autophagy phenotypes together. Calstabin2-deficient mice showed shortened cardiac telomeres alongside activation of AKT/mTOR signalling and impaired autophagy (Yuan et al., 2014). Calorie restriction in middle-aged and old mice was accompanied by increased autophagy, attenuated telomere shortening and improved cardiac remodelling, although the same intervention in young mice had opposite effects (Sheng et al., 2017). In cardiomyocytes exposed to doxorubicin, TRF1 and TRF2 levels fell as senescence developed, and a polyphenol and micronutrient combination that restored mitophagic flux also attenuated the senescent phenotype (Foglio et al., 2023). A recent review likewise treats telomere shortening and diminished autophagy as jointly limiting cardiac regenerative capacity (Ajjan et al., 2026). It should be stressed that in each of these studies the two phenotypes were observed to co-occur; none tested whether the telomere phenotype depends on autophagy by interfering with autophagy genes, so a causal ordering cannot be inferred. We also note that no Mendelian randomisation study with telomere length as exposure and a cardiovascular endpoint as outcome was identified.

The endothelial–cardiomyocyte contrast can be drawn for autophagy generally, and follows from their biology. Cardiomyocytes are terminally differentiated and cannot dilute damaged constituents by division, so they depend on layered mitochondrial quality control in which mitophagy and vesicular mitochondrial ejection are terminal steps; accumulation of dysfunctional mitochondria is a hallmark of the ageing and failing heart (Ravindran and Gustafsson, 2025). Cardiomyocyte-specific failures have been resolved at membrane supply and receptor signalling: in naturally aged mice, fewer ER–mitochondria contacts and reduced PISD activity limit mitochondrial phosphatidylethanolamine, disrupting autophagosomal membrane formation and blocking flux (Hong et al., 2025), while in fatty-acid-oxidation-deficient hearts mitophagy is suppressed despite pronounced mitochondrial stress through impaired PINK1/Parkin signalling and PARL dysregulation, and is restored by deleting USP30 (Sun et al., 2025). Cultured neonatal murine cardiomyocytes entering premature senescence accumulate protein aggregates with declining macroautophagy and altered contractility (Häseli et al., 2020), and myocardial autophagy is bidirectional and dose-dependent under doxorubicin (Wang S. et al., 2025). Endothelial cells, by contrast, are proliferative and senescence-prone, and their dominant described route is receptor-mediated mitophagy: FUNDC1 is reduced in pulmonary hypertension vessels of patients and animals, and inducible endothelial Fundc1 loss alone causes pulmonary hypertension in mice through impaired basal mitophagy, glycolytic reprogramming and senescence, an effect not reproduced by smooth-muscle deletion (Pei et al., 2025); pharmacological activation of SMAD3-dependent PINK1 and LC3 transcription reduces senescence in human aortic endothelial cells (Ding et al., 2025), within a wider endothelial ageing network of Klotho, nitric oxide, FGF21 and sirtuins (Li et al., 2025). These are two separate literature rather than a comparison. No study measures telomere dysfunction and autophagic flux in matched endothelial and cardiomyocyte preparations, so the cell-type difference in telomere-directed autophagy is at present unmeasured; side-by-side iPSC-derived endothelial cells and cardiomyocytes assayed for telomere dysfunction-induced foci, cytosolic telomeric DNA and flux under ATG or receptor knockdown would be the minimum design.

#### 4.3.1 Therapeutic strategies for cardiovascular diseases

Regulating telomeric autophagy through drugs may lead to advancements in cardiovascular disease treatment.

- Autophagy Activators: Drugs such as rapamycin or novel molecules can enhance telomeric autophagy in cardiovascular cells, reducing telomere shortening and improving cardiovascular health. For example, rapamycin can protect cardiac cells by activating autophagy via mTOR inhibition (Kim and Guan, 2015), and thus slows cardiovascular disease progression.
- Antioxidants: The development of cardiovascular diseases is closely linked to oxidative stress (Opresko et al., 2025). The use of antioxidants can reduce oxidative damage in the cardiovascular system, and thus indirectly promotes telomeric autophagy and safeguards cardiovascular health.

## 5 Telomeric autophagy in other diseases

In addition to cancers, neurodegenerative diseases, and cardiovascular diseases, telomeric autophagy is increasingly recognized for its roles in other diseases.

### 5.1 Diabetes and telomeric autophagy

Diabetes is a chronic metabolic disorder associated with cellular aging and telomere shortening. Studies show that diabetic patients have shorter telomeres and impaired autophagy function. Thus, enhancing telomeric autophagy could improve cellular function in diabetic patients and slow disease progression. For instance, increased telomeric autophagy in pancreatic beta cells may help maintain telomere length and function, improving insulin secretion and alleviating diabetes symptoms (Kulkarni et al., 2020).

The most direct evidence available in a diabetic context concerns a shelterin component acting away from telomeres. TIN2 localises both to telomeres and to mitochondria, and in retinal pigment epithelial cells under high glucose it reduced mitophagy and promoted senescence, an effect reversed by rapamycin (Zhang et al., 2024). Three qualifications matter for the present review: the functional pool of TIN2 in that study was mitochondrial, the process suppressed was mitophagy rather than a telomere-directed process, and telomere length and telomere dysfunction were not measured. The finding therefore belongs to the extratelomeric category defined in Section 2.1. In the diabetic kidney, telomere attrition, senescence and autophagic decline have been discussed together as contributors to accelerated ageing, though without evidence establishing which is proximal (Schmitt et al., 2015). We found no study examining telomeres, telomerase or shelterin components in pancreatic islets or beta cells in relation to autophagy, and the earlier claim that enhancing telomere-directed autophagy in beta cells improves insulin secretion is not supported by the literature we identified.

How hyperglycaemia damages the autophagic machinery is defined at several nodes, none of which has been examined in relation to telomeres. The most consistent lesion is a block in degradative capacity rather than in initiation. Human proximal tubular cells exposed to 30 mM glucose, and kidneys of diabetic mice, accumulate LC3-II and SQSTM1 simultaneously—the signature of impaired clearance—through induction of TXNIP (Huang et al., 2014); in podocytes, high glucose and advanced glycation end products block autophagic flux by way of lysosomal dysfunction *in vitro* and *in vivo*, a defect corrected by tunnelling-nanotube-mediated exchange of autophagosomes and lysosomes (Barutta et al., 2023). Prolonged hyperglycaemia combined with failing lysosomes converts autophagy induction into autophagic stress (Zheng et al., 2020), and restoring TFEB activity re-establishes autophagosome maturation, autophagosome–lysosome fusion and lysosomal degradation (Li et al., 2026). Selective pathways are affected individually: ER-phagy fails through loss of PACS-2 and of the receptor FAM134B (Yang et al., 2023), microglial lipophagy is dampened so that lipid droplets accumulate (Li Q. et al., 2023), and neuronal mitophagy is suppressed because high-glucose-derived reactive oxygen species inhibit AMPK and activate a RELA–HDAC8 complex that represses PRKN transcription (Cho et al., 2024). In rodent cardiomyocytes, high glucose reduces mitophagic flux and lowers PINK1–Parkin expression (Kobayashi et al., 2020), (Yang et al., 2024). The direction is not uniform across cell types: in HUVECs and in the retinal vasculature of db/db mice, high glucose instead stimulates autophagosome synthesis, and endothelial ATG7 deletion indicated that this autophagy is detrimental (Niu et al., 2019). What is absent is any telomeric measurement. No study in this evidence base asks whether hyperglycaemia alters the generation or clearance of telomeric DNA damage, whether cytosolic telomeric fragments accumulate once lysosomal capacity fails, or whether the cGAS–STING route described in Section 2 is engaged—experiments that would need telomere dysfunction-induced foci, cytosolic telomeric DNA and autophagic flux measured together in the same high-glucose-exposed cells.

### 5.2 Liver diseases and telomeric autophagy

The liver is one of the most metabolically active organs, and its health is closely tied to telomere length and autophagy function. Patients with liver diseases (e.g., cirrhosis and liver cancer) often exhibit shorter telomeres and impaired autophagy function in liver cells (Meng et al., 2015). Enhancing telomeric autophagy may improve liver cell function and slow the progression of liver diseases (Penrice and Simonetto, 2020). Moreover, increased telomeric autophagy could support liver regeneration and thus enhance disease prognosis.

The clearest link between telomeres and liver disease comes from telomere biology disorders. Short telomere syndromes produce hepatic as well as haematological and pulmonary manifestations (Penrice and Simonetto, 2020), and in a National Cancer Institute cohort of 231 patients, severe liver disease was more frequent in autosomal recessive, X-linked and TINF2-associated forms (Niewisch et al., 2022). In a multicentre series of 132 patients with germline telomere-related gene mutations compared with 1,190 matched controls, liver involvement was strongly over-represented (Sidali et al., 2024), and short telomeres were found in the majority of patients with idiopathic porto-sinusoidal vascular disorder (Coukos et al., 2024).

The epidemiological evidence available for these two settings concerns telomere length, and is summarised here on those terms. For type 2 diabetes, a meta-analysis of nine cohorts (5,759 cases, 6,518 controls) gave an OR of 1.29 (95% CI 1.11–1.50) for shortened telomeres, falling to 1.12 (1.00–1.25) once the three cohorts responsible for the heterogeneity were removed (Zhao et al., 2013); pooling three prospective cohorts (6,991 participants, 2,011 incident events) gave a relative risk of 1.31 (1.07–1.60) (Willeit et al., 2014). Associations with progression are firmer than those with incidence: among 5,349 Chinese patients followed for a mean of 8.8 years, shorter baseline LTL predicted progression to insulin requirement (1.69-fold per 1-kb decrease in absolute length, 1.35–2.11), an association supported by two-sample Mendelian randomisation (1.38-fold per genetically predicted unit decrease, 1.12–1.70) (Cheng et al., 2022a), and in 4,085 patients followed for 14.1 years it predicted incident end-stage kidney disease (adjusted HR 1.11 per unit decrease, 1.03–1.19) (Cheng et al., 2022b). For incidence, by contrast, genetic approaches have been null: in the Women’s Health Initiative (1,675 incident cases, 2,382 controls) neither measured LTL (OR 1.00 per 1-kb increment, 0.90–1.11 in White participants) nor genetic instruments predicted diabetes (P = 0.52) (You et al., 2012). Liver disease follows the same pattern. NHANES (n = 7,072) found no overall association between LTL and liver disease, and within the liver-disease cohort the association between shorter LTL and all-cause mortality did not reach significance (HR 1.22, 95% CI 0.99–1.51) (Rattan et al., 2022); over 21 years in 847 middle-aged hypertensive adults, however, the shortest LTL tertile was associated with prevalent non-alcoholic fatty liver disease relative to the longest (HR 2.03, p = 0.007) (Korkiakoski et al., 2022). The largest effects come not from population variation but from germline defects: liver disease affected 95 of 132 carriers of telomere-related gene mutations (72%) versus 80 of 396 matched community controls (20%), an OR of 12.9 (7.8–21.3), with 5-year transplantation-free survival of 69% (Sidali et al., 2024). Consistently, among 1,214 patients with alcohol-related cirrhosis and hepatocellular carcinoma versus 1,866 without, the TERT allele rs2242652(A) — associated with longer leucocyte telomeres—was protective (OR 0.61, 0.52–0.70) (Buch et al., 2023).

Autophagy is separately implicated in hepatic ageing, although not always in the protective direction often assumed: DRAM1-mediated autophagy in mouse liver and mesenchymal stem cells was pro-senescent rather than protective, and did not downregulate SQSTM1/p62 as bulk autophagy does (Yang J. et al., 2025). These two bodies of work do not intersect. Among the studies we identified, those linking telomeres to liver disease did not measure autophagy, and those examining hepatic autophagy did not measure telomeres; none measured both. They also differ in kind, the first being largely clinical and genetic and the second largely experimental. Combining them to conclude that telomere-directed autophagy is impaired in liver disease would assert something neither supports, and we do not draw that conclusion here.

Table 2 summarises, for each disease discussed in Sections 4 and 5, what is known about telomeres, what is known about autophagy, and whether the two have been examined together. Presented in this form, a pattern emerges that is not evident when the diseases are read one at a time: cancer is the only setting in which the two lines of evidence have been experimentally joined, and it is also the only setting in which the direction of the effect has been shown to depend on context. Elsewhere, the proposition that telomere-directed autophagy is impaired rests on the coincidence of two separate literature rather than on any study that measured both.

**TABLE 2:** Evidence status of the telomere–autophagy relationship across diseases. The fourth column is the one that distinguishes the rows: only in cancer have telomeric and autophagic endpoints been measured in the same system with genetic perturbation of the autophagy machinery. For the remaining diseases the model of impaired telomere-directed autophagy is an inference drawn from two separate literature, and we identify it as such rather than presenting it as a shared mechanism.

Disease	Telomere evidence	Autophagy evidence	Have the two been examined together?	Status of the “impaired telomeric autophagy” model
Cancer	Telomerase reactivation and ALT sustain proliferation; telomere crisis acts as a barrier (Chakravarti et al., 2021; Nassour et al., 2019; Hayashi et al., 2015)	Macroautophagy executes death in crisis; pro-survival in ALT-positive cells (Nassour et al., 2019; Barroso-González et al., 2019)	Yes. Both measured in the same systems, with genetic perturbation of autophagy (Nassour et al., 2019; Barroso-González et al., 2019)	Supported, and context-dependent: tumour-suppressive during crisis, pro-survival once established
Neurodegeneration	Leukocyte telomere length associations are inconsistent: U-shaped in one cohort (Fani et al., 2020), null longitudinally in another (Nudelman et al., 2019)	Autophagic decline is well documented (Kodali et al., 2025; Kulkarni et al., 2020)	No. No study identified measured both in neural tissue; all human telomere data are from blood (Konopka, 2025)	Not established. Neurons are post-mitotic, so the mechanism of telomere attrition is itself unclear
Cardiovascular disease	Leukocyte telomere length associates with disease in some cohorts but not others (Drury et al., 2012; Hunt et al., 2015; Fernández-Alvira et al., 2016; Bountziouka et al., 2022)	Selective autophagy contributes to cardiometabolic homeostasis (Jeong et al., 2019)	Co-occurrence only. Four studies report both phenotypes (Yuan et al., 2014; Sheng et al., 2017; Foglio et al., 2023; Ajjan et al., 2026); none tested dependence	Not established. No study interfered with autophagy and asked whether the telomere phenotype was lost
Diabetes and metabolic disease	Shorter telomeres reported in patients; no islet or beta-cell data identified	TIN2 restrains mitophagy in retinal pigment epithelium under high glucose (Zhang et al., 2024)	Only extratelomerically. In (Zhang et al., 2024) the functional pool was mitochondrial and telomeres were not measured	Not established. The relevant tissue has not been examined
Liver disease	Telomere biology disorders produce hepatic manifestations, with cohort and multicentre data (Penrice and Simonetto, 2020; Niewisch et al., 2022; Sidali et al., 2024; Coukos et al., 2024)	DRAM1-mediated autophagy is pro-senescent in liver, not protective (Yang C. et al., 2025)	No. The telomere and autophagy literature do not intersect; none measured both	Not established. The two bodies of work also differ in kind, being clinical and experimental respectively

## 6 Mechanisms of telomeric autophagy dysfunction

Telomeric autophagy is a vital mechanism in the autophagy system for addressing telomere damage. However, its function can be impaired in various physiological and pathological conditions, directly affecting telomere length and cellular functionality. Understanding the mechanisms underlying telomeric autophagy dysfunction is a key to investigating the development and progression of aging and diseases.

### 6.1 Abnormalities in telomere-protective proteins

The roles of TRF1 and TRF2 are described in Section 2.1 and are not repeated here. This section considers only how their dysregulation has been linked to altered autophagy (Li K. Q. et al., 2023; Muraki and Murnane, 2018; Sarek et al., 2015).

- Overexpression of TRF2: Excessive TRF2 expression may inhibit the initiation of telomeric autophagy by overprotecting telomeres, preventing the clearance of damaged telomeres.
- Downregulation of TRF1: Downregulation of TRF1 can disrupt telomere recognition and handling, impairing the telomeric autophagy process.

### 6.2 Mutations or abnormal expression of ATGs

The autophagy process involves a complex interplay of multiple genes. Mutations or abnormal expression of these ATGs can impair telomeric autophagy function.

- ATG Mutations: The best-characterised example in human disease is the ATG16L1 T300A coding variant, in which a single threonine-to-alanine substitution renders the protein susceptible to caspase-mediated cleavage and reduces selective autophagy specifically, leaving bulk autophagy comparatively intact (Lassen et al., 2014). The variant is associated with susceptibility to Crohn’s disease (Simovic et al., 2024). It is worth noting why this example is instructive here: it shows that a point mutation can impair the selective arm of the pathway while sparing the bulk arm, which is precisely the kind of genetic dissociation that would be needed to establish a telomere-directed arm. No comparable variant has been linked to telomere maintenance. More broadly, mutations in autophagy genes can prevent proper autophagosome formation and thereby impair autophagic degradation (Mizushima and Komatsu, 2011; Klionsky et al., 2021).
- LC3 and p62 Abnormalities: LC3 is required for autophagosome membrane elongation and p62/SQSTM1 serves as a cargo receptor linking ubiquitinated substrates to LC3 (Bitto et al., 2014; Huang et al., 2023; Clausen et al., 2010; Mizushima and Komatsu, 2011). Abnormal expression or dysfunction of either protein impairs autophagic degradation generally; a specifically telomere-directed consequence has not been demonstrated.

### 6.3 mTOR signaling pathway abnormalities

As described in Section 2.3, mTOR is a key negative regulator of autophagy. Abnormal mTOR activity can directly impair autophagy (Collins et al., 2021; Kim and Guan, 2015). mTORC1 integrates nutrient and growth-factor availability and, when active, phosphorylates and inhibits the ULK1 complex, suppressing autophagy initiation; conversely, energetic stress activates AMPK, which relieves this inhibition (Mizushima and Komatsu, 2011). Because mTOR signalling is constitutively elevated in many tumours and rises with age in several tissues, the pathway has been an obvious candidate for linking autophagic decline to telomere maintenance. It should be noted, however, that the evidence connecting mTOR activity to telomere status is indirect: studies have measured either autophagic markers or telomere length, rarely both, and none that we identified established that an mTOR-dependent change in telomere length requires an intact autophagy pathway. Two further points address the specificity of this pathway. First, mTOR functions within two complexes with distinct compositions and outputs, and it is mTORC1 rather than mTORC2 that directly restrains autophagy initiation through the ULK1 complex; the activation of mTORC1 by RHEB and its inhibition by PRAS40 have been resolved structurally (Yang et al., 2017). Second, cancer-associated MTOR mutations are frequently hyperactivating and confer sensitivity to mTOR inhibitors (Grabiner et al., 2014), which provides a genetic route to constitutive suppression of autophagy in tumours. Whether either observation extends to telomere-directed autophagy has not been examined.

- Excessive Activation of mTOR: Overactivation of mTOR inhibits autophagy (Collins et al., 2021; Kim and Guan, 2015), including telomeric autophagy. Continuous activation of the mTOR pathway in cancer cells may lead to reduced autophagy, affecting telomeric autophagy.

### 6.4 Signalling, oxidative and lysosomal contributions to impaired autophagy

p53, a critical tumor suppressor, regulates cell cycle, autophagy, and apoptosis (Maiuri et al., 2010; Mrakovcic and Fröhlich, 2018; Kruse and Gu, 2009). p53 status strongly influences the cellular outcome of telomere dysfunction: in p53-competent cells, dysfunctional telomeres trigger senescence or apoptosis, whereas loss of p53 permits survival and propagation of genomically unstable cells (Hayashi et al., 2015). A specific requirement for p53 in telomere-directed autophagy, however, has not been demonstrated.

Beyond signalling, two further routes converge on the same outcom. Oxidative stress and DNA damage are significant triggers for telomeric autophagy dysfunction (Opresko et al., 2025). Excessive reactive oxygen species (ROS) accumulation can damage telomeres, triggering telomeric autophagy. Moreover, prolonged oxidative stress may overwhelm cellular repair mechanisms, impairing telomeric autophagy.

Lysosomal dysfunction affects the fusion of autophagosomes with lysosomes, thereby impairing autophagic degradation in general (Mizushima and Komatsu, 2011; Klionsky et al., 2021). Whether this impairment extends specifically to telomere-associated autophagy has not been tested directly.

## 7 Interventions to enhance telomeric autophagy

Enhancing telomeric autophagy through various interventions may improve cellular health by preventing telomere shortening.

### 7.1 Pharmacological, genetic, nutritional and lifestyle approaches

- Rapamycin: Rapamycin activates autophagy by inhibiting the mTOR pathway (Kim and Guan, 2015), potentially delaying telomere shortening.
- Metformin: Known for activating AMPK, metformin indirectly promotes autophagy (Kulkarni et al., 2020), including telomeric autophagy.
- Antioxidants: Antioxidants reduce ROS accumulation and alleviate oxidative stress, which is a recognised source of telomeric DNA damage (Opresko et al., 2025). An effect on telomere-associated autophagy has not been measured.

Genetic approaches have also been proposed. Gene editing techniques such as CRISPR/Cas9 and RNA interference (RNAi) can modulate the expression of telomere-protective or autophagy-related genes (Klionsky et al., 2021). Such approaches have been used to establish dependence on the core autophagy machinery in other settings, but have not been applied to test whether telomeres are autophagic cargo.

- Non-pharmacological interventions are likewise under consideration. Intermittent Fasting: Metabolic regulation during fasting activates autophagy (Mizushima and Komatsu, 2011). We are not aware of studies in which telomere endpoints were measured alongside autophagic flux under fasting regimens.
- Exercise: Regular physical activity enhances autophagy and improves cellular health.

### 7.2 Emerging molecules and compounds

Resveratrol has been suggested to promote autophagy and is of interest in aging-related and degenerative disease (Mizushima and Komatsu, 2011). Its effect on telomeres has not been measured directly. We note that sirolimus, listed in an earlier version of this section as a separate emerging compound, is an alternative name for rapamycin and is therefore discussed in Section 7.1 rather than here.

A caveat applies to this entire section. The interventions above are general modulators of autophagy or of senescent-cell viability; none has been shown to act selectively on telomere-associated autophagy, and for several of them (antioxidants, gene editing, intermittent fasting and exercise) we are not aware of studies that measured telomere endpoints alongside autophagic flux in the same experiment. Their effects on telomeres should therefore be regarded as extrapolation rather than demonstrated mechanism. We explicitly acknowledge that the therapeutic targeting of telomere-associated autophagy remains an emerging and insufficiently explored area, and that establishing it as a tractable strategy will first require confirming that the pathway itself exists in the selective form proposed.

The agents summarised in Table 3 act at four distinct nodes of the pathway. The first and largest group works upstream, at the nutrient-sensing kinases. Rapamycin, the prototype, does not inhibit mTOR directly: it first binds the immunophilin FKBP12, and the resulting drug–protein complex engages the FKBP12–rapamycin-binding (FRB) domain of mTOR, allosterically restraining mTORC1 and relieving its repression of autophagy initiation (Kim and Guan, 2015; Yang et al., 2017). This mechanism explains both rapamycin’s selectivity and its limitations: it is an incomplete inhibitor that fails to abolish multisite 4E-BP1 phosphorylation and leaves mTORC2 untouched (Willems et al., 2012; Liao et al., 2015). The rapalogs everolimus and temsirolimus share this allosteric mechanism and the same ceiling, and compensatory mTORC2 signalling has been proposed as a route to resistance (Zhang et al., 2013). Second-generation ATP-competitive inhibitors—AZD8055, AZD 2014, KU0063794 and OSI-027 — were designed to close that gap by occupying the mTOR catalytic site, blocking both complexes and abrogating PI3K/AKT feedback; all induce autophagy more completely than rapalogs (Vakana et al., 2010; Willems et al., 2012; Huo et al., 2014; Liao et al., 2015; Chen and Zhou, 2020). A second group acts through AMPK, which inhibits mTORC1 and phosphorylates ULK1 directly, coupling energy status to autophagy initiation (Egan et al., 2011; Hu et al., 2021); metformin and resveratrol operate here (Ren et al., 2020; Tai et al., 2022; Xia et al., 2024). A third group bypasses mTOR entirely and works at the lysosome and its transcriptional programme: trehalose activates TFEB through low-grade lysosomal stress and calcineurin (Rusmini et al., 2019; Jeong et al., 2021), while spermidine acts through eIF5A hypusination to favour TFEB translation, an effect abolished in Atg5-deficient hearts (Eisenberg et al., 2016; Hofer et al., 2024a). Finally, chloroquine, hydroxychloroquine and bafilomycin A1 block flux at the degradative step, by distinct and non-interchangeable mechanisms (Mauthe et al., 2018). The converse asymmetry is equally striking: the studies that did measure telomere length in relation to antioxidant exposure—a Mendelian randomisation analysis of micronutrient levels (Zhu et al., 2026), dietary and supplementation intervention studies (Corina et al., 2019; Nonsa-Ard et al., 2022), and work in a non-mammalian species (Kim and Velando, 2015) — did not report autophagic markers. The two literature have been built by different communities asking different questions, and they have not met. Figure 3 maps these agents onto the pathway and marks the evidence strength of each link.

**TABLE 3:** Pharmacological and non-pharmacological modulators of autophagy, with their molecular targets and mechanisms of action. The final column is the one that matters for this review. Across the studies cited here, most agents have never been tested with a telomere endpoint at all, and for the remainder the telomere-related observation is indirect or heavily qualified. No agent in this table has been shown to act selectively on telomere-associated autophagy.

Agent	Class	Molecular target	Mechanism of action on autophagy	Evidence for an effect on telomeres
Rapamycin (sirolimus)	First-generation, antibiotic allosteric mTOR inhibitor (Chen and Zhou, 2020)	mTORC1 (via FKBP12; the drug–immunophilin complex engages the FKBP12–rapamycin-binding (FRB) domain of mTOR) (Yang et al., 2017)	Allosteric inhibition of mTORC1 relieves mTORC1-mediated repression of autophagy initiation; mTOR is the best-characterised pharmacological node for autophagy induction (Kim and Guan, 2015; Vakifahmetoglu-Norberg et al., 2015). Inhibition is incomplete: rapamycin does not fully block multisite 4E-BP1 phosphorylation and does not inhibit mTORC2 [85,91]. Tumours carrying hyperactivating *MTOR* mutations show heightened rapamycin sensitivity (Grabiner et al., 2014)	Indirect and heavily qualified. Micro-dose rapamycin (below the TORC1-inhibiting concentration, i.e., not acting through the autophagy-inducing mechanism) prevented death of senescent human fibroblasts induced by telomere dysfunction (Qi et al., 2014). In *Drosophila*, a rapamycin-supplemented diet was studied alongside telomere-maintenance gene expression (*dHeT-A*, *dTahre*, *dTart*), but those transcript changes were attributed to iron chelation rather than to rapamycin (Faruk et al., 2025a; Faruk et al., 2025b). No study cited here measured telomere length or telomeric DNA content under rapamycin
Everolimus	Rapalog (allosteric mTORC1 inhibitor); approved in renal cell carcinoma (Zhang et al., 2013)	mTORC1	Same allosteric mechanism as rapamycin; inhibits mTORC1 but not mTORC2, and compensatory mTORC2 activation has been proposed as a resistance mechanism (Zhang et al., 2013). In the randomised phase 2 CLEVER trial, everolimus alone or with hydroxychloroquine reduced disseminated tumour cell burden in breast cancer survivors (DeMichele et al., 2025)	No telomere endpoint measured (CLEVER endpoints were DTC clearance and recurrence-free survival) (Zhang et al., 2013; DeMichele et al., 2025)
Temsirolimus	Rapalog (allosteric mTORC1 inhibitor); approved in renal cell carcinoma (Zhang et al., 2013)	mTORC1	Allosteric mTORC1 inhibition. In renal cell carcinoma xenografts it matched a dual mTORC1/2 inhibitor for tumour growth control, apparently through additional effects on the tumour microenvironment (reduced angiogenesis, lower VEGF/PDGF) (Zhang et al., 2013); in spinal cord injury it reduced neuroinflammatory markers comparably to rapamycin (Cordaro et al., 2017)	No telomere endpoint measured (Zhang et al., 2013; Cordaro et al., 2017)
AZD8055, AZD 2014 (vistusertib), KU0063794, OSI-027	Second-generation ATP-competitive dual mTORC1/mTORC2 inhibitors (Chen and Zhou, 2020)	mTOR kinase active site (both complexes)	Compete with ATP at the mTOR catalytic site, so they block mTORC1 more completely than rapalogs (full multisite 4E-BP1 dephosphorylation) and block mTORC2, abrogating the PI3K/AKT feedback loop; all four induce autophagy — AZD8055 in AML (Willems et al., 2012), AZD2014 in hepatocellular (Liao et al., 2015) and colorectal carcinoma (Huo et al., 2014), KU0063794 in renal cell carcinoma (Zhang et al., 2013) and after spinal cord injury (Cordaro et al., 2017), OSI-027 in BCR-ABL^+^ leukaemia (Vakana et al., 2010). The induced autophagy is cytoprotective in several models: blocking it with chloroquine or hydroxychloroquine converts the response to apoptosis (Vakana et al., 2010; Huo et al., 2014)	No telomere endpoint measured in any of these studies (Vakana et al., 2010; Willems et al., 2012; Zhang et al., 2013; Huo et al., 2014; Liao et al., 2015; Cordaro et al., 2017)
Metformin	Biguanide; indirect AMPK activator/caloric-restriction mimetic	AMPK–mTOR axis; AMPK–SIRT1–FoxO1; PINK1–PRKN (Ren et al., 2020; Han et al., 2021)	Activates AMPK, which inhibits mTORC1 and directly phosphorylates ULK1, thereby restoring autophagic flux. Metformin restored autophagic flux and suppressed senescence in vascular smooth muscle cells (Tai et al., 2022), enhanced autophagy and relieved oxidative stress in diabetic kidney via AMPK/SIRT1–FoxO1 [117], and rescued PINK1/PRKN-dependent mitophagy in diabetic renal tubule — an effect abolished by the AMPK inhibitor compound C or *Pink1* siRNA (Han et al., 2021); reviewed as a geroprotector acting through AMPK–mTOR and autophagy (Ala and Ala, 2021; Zhang et al., 2025)	Assertion only, no primary measurement. A narrative review lists “lowering telomere attrition” among metformin’s effects on the hallmarks of ageing (Kulkarni et al., 2020), but none of the primary metformin–autophagy studies cited here measured telomeres (Ren et al., 2020; Tai et al., 2022; Han et al., 2021)
Direct AMPK activators (AMPK–ULK1 axis)	Energy-stress mimetics/AMPK agonists	AMPK → ULK1 (ATG1 orthologue); AMPK–MFN2 [82,122]	AMPK phosphorylates ULK1/ULK2 at conserved sites; a ULK1 mutant that cannot be phosphorylated by AMPK fails to support mitophagy, causing p62 accumulation and loss of mitochondrial homeostasis during starvation (Egan et al., 2011). Under energy stress AMPK also translocates to mitochondria-associated ER membranes and binds MFN2 to sustain autophagy (Hu et al., 2021); the AMPK–ULK1–autophagy axis is pharmacologically addressable *in vivo* (Zhang et al., 2022; Han et al., 2021)	No telomere endpoint measured (Egan et al., 2011; Hu et al., 2021; Zhang et al., 2022)
Spermidine	Natural polyamine; caloric-restriction mimetic (Hofer et al., 2022)	eIF5A hypusination → TFEB translation; ATG5-dependent machinery (Eisenberg et al., 2016; Hofer et al., 2024a)	Induces autophagy through the polyamine–hypusination axis: spermidine-dependent hypusination of eIF5A favours translation of the pro-autophagic transcription factor TFEB and raises autophagic flux (Hofer et al., 2024a; Hofer et al., 2024b). Its cardioprotective and lifespan effects are autophagy-dependent — they are lost in mice lacking cardiomyocyte Atg5 [100]. An endogenous spermidine surge is also required for rapamycin-induced autophagy and longevity (Hofer et al., 2024b)	Measured, and positive. Six months of oral spermidine in aged mice attenuated multiple ageing phenotypes, and this protection was associated with decreased telomere attrition (Wirth et al., 2021). This is an association reported within the study, not a demonstration that spermidine acts on telomeres, and telomeric autophagy was not assessed
Trehalose	Natural disaccharide; mTOR-independent autophagy inducer	TFEB (via lysosomal perturbation and PPP3/calcineurin) (Rusmini et al., 2019; Jeong et al., 2021)	Accumulates in the endolysosomal system, producing transient lysosomal enlargement, membrane permeabilisation and a mild rise in lysosomal pH; this low-grade lysosomal stress activates PPP3/calcineurin, dephosphorylates TFEB and drives its nuclear translocation, upregulating lysosomal and autophagy genes (Rusmini et al., 2019; Jeong et al., 2021)	Telomerase, not telomeres. Trehalose (2% for 28 days) was used as an autophagy activator in TERT-mutant rats and restored NO-mediated flow-mediated dilation, placing autophagy downstream of TERT (Hughes et al., 2025). Telomere length was not measured
Resveratrol	Polyphenol; antioxidant and AMPK activator	AMPK (Xia et al., 2024)	Promotes AMPK phosphorylation, thereby activating autophagy, lowering ROS, inhibiting apoptosis and restoring cell-cycle progression in UVA-irradiated human skin fibroblasts and in mouse skin (Xia et al., 2024). Grouped with metformin and rapamycin as a caloric-restriction mimetic, acting on sirtuin signalling rather than on mTOR directly (Speakman and Mitchell, 2011)	No telomere endpoint measured (Speakman and Mitchell, 2011; Xia et al., 2024)
N-acetylcysteine (NAC)	Thiol antioxidant/glutathione precursor	Cellular ROS pool (upstream of ROS-dependent AMPK–mTOR signalling) (Guo H. et al., 2022)	Suppresses, rather than induces, oxidant-driven autophagy: in copper-overloaded testis and GC-1 spg cells, NAC attenuated CuSO_4_-induced autophagy *in vivo* and *in vitro* and reversed activation of the AMPK–mTOR pathway (Guo H. et al., 2022). Its established clinical use is as the antidote for acetaminophen overdose, replenishing hepatic glutathione, in a setting where autophagic removal of damaged mitochondria is a parallel modulator of injury (Yan et al., 2018; Ramachandran and Jaeschke, 2019)	No telomere endpoint measured (Ramachandran and Jaeschke, 2019; Guo H. et al., 2022)
Mitochondria-targeted antioxidants (MitoQ, SkQ1, MitoTEMPO)	Lipophilic triphenylphosphonium cation–antioxidant conjugates (Oyewole and Birch-Machin, 2015; Zinovkin and Zamyatnin, 2019; Wu and Wu, 2026)	Mitochondrial matrix ROS	Accumulate in mitochondria driven by membrane potential and scavenge ROS at the source (Oyewole and Birch-Machin, 2015; Wu and Wu, 2026). Effects on the autophagy–lysosome system are indirect and only partly characterised in these sources: MitoTEMPO reversed premature senescence of ovariectomised-rat BMSCs by reinstating lysosomal function and suppressing the mitochondrial unfolded protein response (Li J. et al., 2024); mitochondrial oxidative damage is itself the signal for BNIP3/NIX-dependent mitophagy (Sulkshane et al., 2021). MitoQ improved cognition and extended lifespan in 3xTg-AD mice (Young and Franklin, 2019) and improved endothelial function in a randomised crossover trial in healthy older adults (Rossman et al., 2018), but autophagic flux was not an endpoint in either	No telomere endpoint measured (Rossman et al., 2018; Young and Franklin, 2019; Li J. et al., 2024; Wu and Wu, 2026)
Dasatinib + quercetin (D + Q)	Senolytics (quercetin is also a flavonoid antioxidant)	Senescent-cell anti-apoptotic pathways (SCAPs) (Hickson et al., 2019)	Not autophagy modulators in the strict sense: they selectively eliminate senescent cells by transiently disabling the pro-survival networks that protect those cells from their own pro-apoptotic environment (Hickson et al., 2019). Because senescence is a downstream consequence of telomere attrition, D + Q acts on the cellular product of telomere dysfunction rather than on autophagic flux. Clinically tested in diabetic kidney disease (Hickson et al., 2019), idiopathic pulmonary fibrosis (Justice et al., 2019; Nambiar et al., 2023), Alzheimer’s disease (Gonzales et al., 2022; Gonzales et al., 2023) and postmenopausal bone loss (Farr et al., 2024)	No telomere endpoint measured in any of these trials; senescence was quantified by p16/SA-β-gal and SASP markers, not by telomere length (Hickson et al., 2019; Nambiar et al., 2023; Gonzales et al., 2023; Farr et al., 2024)
Chloroquine/hydroxychloroquine	4-aminoquinoline lysosomotropic autophagy inhibitors; the only FDA-approved autophagy inhibitors in clinical trials (Mauthe et al., 2018)	Lysosome/autophagosome–lysosome fusion	Block autophagic flux principally by impairing autophagosome–lysosome fusion rather than by acidity or degradative capacity *per se*, and additionally cause autophagy-independent disorganisation of the Golgi and endolysosomal system — including in kidney and intestine of HCQ-treated mice — so they are not clean surrogates for other late-stage lysosomal inhibitors (Mauthe et al., 2018; Schrezenmeier and Dörne, 2020). Used to convert cytoprotective drug-induced autophagy into cell death (Vakana et al., 2010; Huo et al., 2014; Cocco et al., 2022) and tested clinically with everolimus (DeMichele et al., 2025)	No telomere endpoint measured (Mauthe et al., 2018; Schrezenmeier and Dörne, 2020; Cocco et al., 2022; DeMichele et al., 2025)
Bafilomycin A1	Vacuolar-type ATPase inhibitor; late-stage lysosomal inhibitor (tool compound only, no clinical use)	Lysosomal acidification and degradative activity (Mauthe et al., 2018)	Standard flux-clamping reagent: unlike chloroquine, its block operates at the level of lysosomal acidity/degradative activity, which is why the two drugs are not interchangeable (Mauthe et al., 2018). Routinely used as the flux-blocking arm of LC3-II turnover assays in the consensus autophagy guidelines (Klionsky et al., 2021), and applied as such across the studies tabulated here (Li J. et al., 2024; Brun et al., 2022; Hao et al., 2025)	No telomere endpoint measured (Klionsky et al., 2021; Mauthe et al., 2018)
T-oligo (telomere 3′overhang-mimetic oligonucleotide)	Telomere-derived experimental agent	mTOR and STAT3 [78]	The only agent in this table whose stimulus originates at the telomere: T-oligos mimic telomere-loop disruption and induce non-apoptotic autophagy in malignant glioma cells by inhibiting both mTOR and STAT3; rapamycin and the STAT3 inhibitor AG490 sensitise cells to T-oligos by augmenting autophagy (Yokoyama et al., 2007)	Inverted case. The telomeric sequence is the *stimulus*, not the endpoint — endogenous telomere length and telomeric chromatin turnover were not measured (Yokoyama et al., 2007)

**FIGURE 3:**
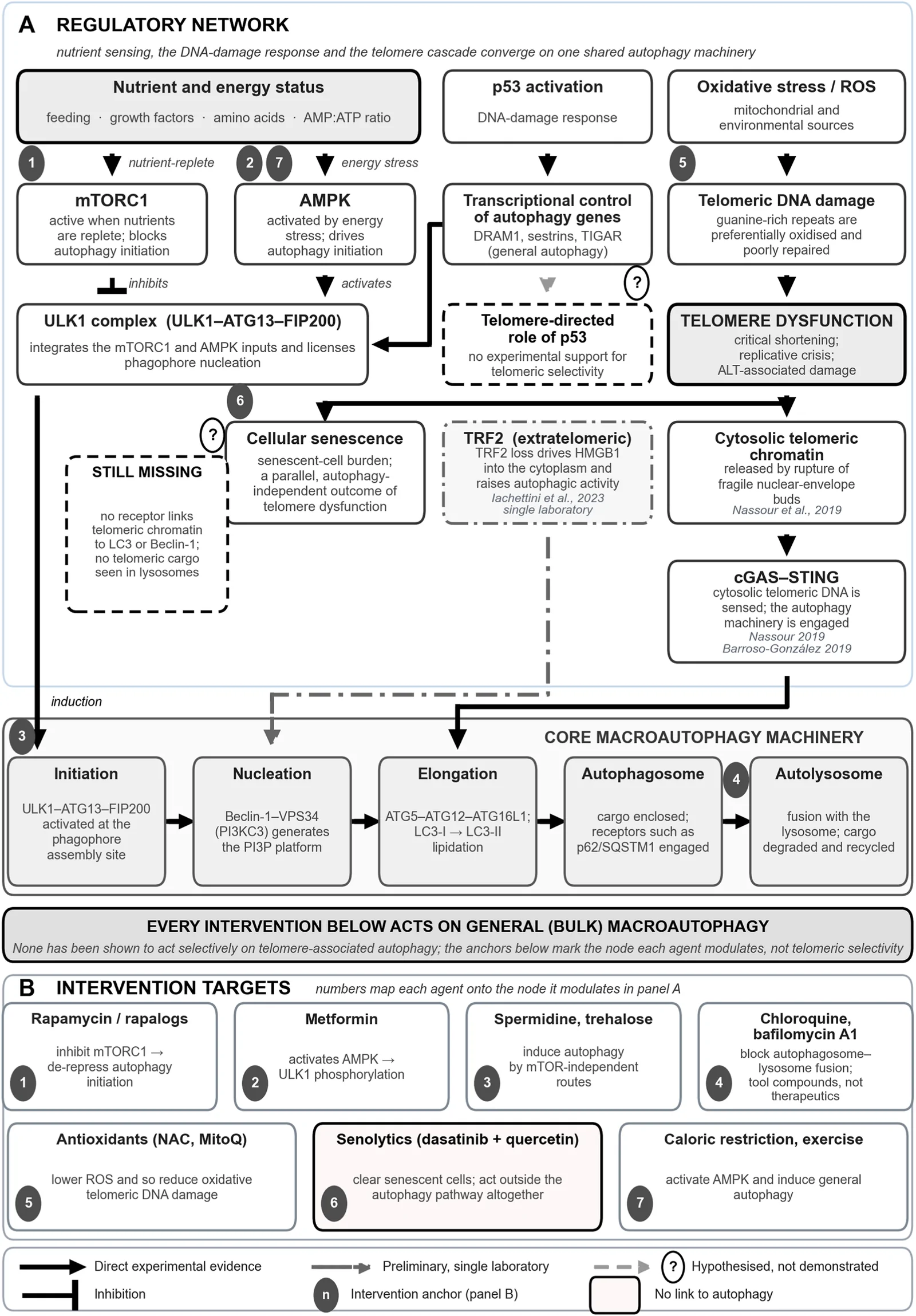
Regulatory network and intervention targets linking telomere biology to macroautophagy. **(A)** Nutrient and energy status signals through mTORC1 (inhibitory) and AMPK (stimulatory) to the ULK1-ATG13-FIP200 complex. p53 drives transcription of autophagy genes, but no evidence supports a telomere-directed role. Oxidative stress promotes telomeric DNA damage and telomere dysfunction; dysfunctional telomeres yield cytosolic telomeric chromatin that is sensed by cGAS-STING and engages the autophagy machinery. Telomere dysfunction also drives senescence, an autophagy-independent outcome. TRF2 modulates autophagy via HMGB1, an extratelomeric function reported by a single laboratory. Line style encodes evidence strength: solid, direct experimental evidence; dash-dot, preliminary single-laboratory evidence; dashed with a question mark, hypothesised or untested. **(B)** Numbered anchors map each agent onto the node it modulates. Every agent shown acts on general (bulk) macroautophagy; none has been shown to act selectively on telomere-associated autophagy.

## 8 Conclusion

The autophagic response to telomere dysfunction is now established as a component of the cellular reaction to telomere damage, and is best characterised in replicative crisis, where it acts through cytosolic telomeric chromatin and cGAS-STING signalling (Nassour et al., 2019; Barroso-González et al., 2019; Nassour et al., 2023). Whether telomeric chromatin is additionally a selective autophagic cargo remains an open question, and resolving it is the necessary first step before therapeutic targeting of this process can be seriously pursued. Progress will depend on studies that measure telomeric and autophagic endpoints in the same system and that test dependence on the core machinery (Klionsky et al., 2021). Figure 4 summarises this position as a timeline, setting the milestones that have been reached at the interface of telomere biology and autophagy against those that have not.

**FIGURE 4:**
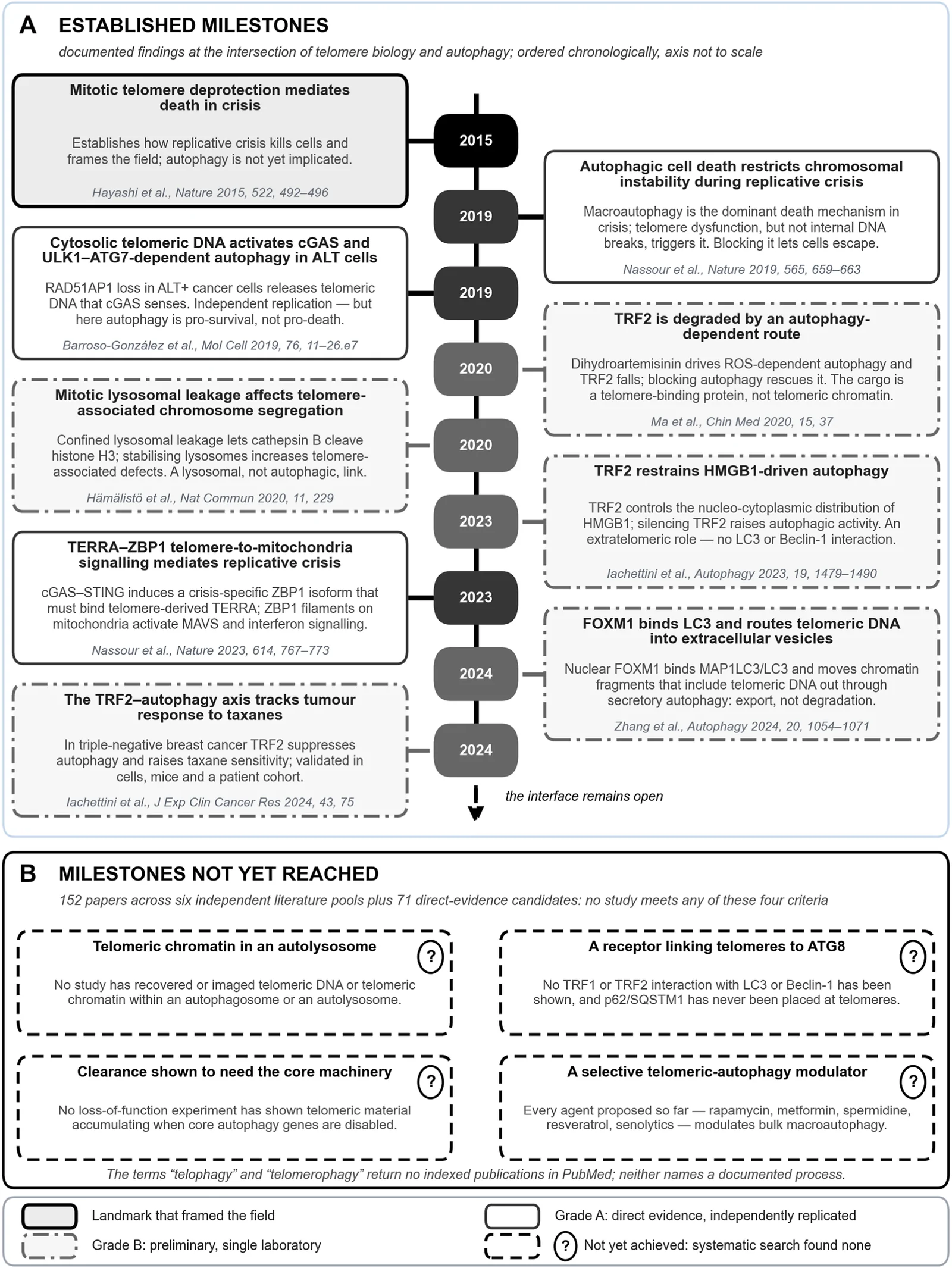
Milestones at the interface of telomere biology and autophagy, and the milestones not yet reached. **(A)** Findings that have been reported, arranged chronologically and shaded by evidence grade: solid markers denote results replicated by independent groups, dash-dot markers denote single-laboratory findings. **(B)** Four results that would be required to establish telomere-selective autophagy as a pathway, none of which has been reported: recovery of telomeric chromatin from within an autolysosome; identification of a receptor bridging telomeric chromatin to an ATG8-family protein; demonstration that clearance of telomeric material depends on the core machinery; and a modulator acting selectively on this process. The contrast between the two panels is the position this review arrives at: an active interface with a well-supported signalling arm and an unestablished degradative arm.
